# Supplementary material for: Improving CPAP Adherence for Obstructive Sleep Apnea: A Practical Application Primer on CPAP Desensitization
Source: MedEdPORTAL. 2020 Sep 15;16:10963. doi: 10.15766/mep_2374-8265.10963 (PMC7499811; doi:10.15766/mep_2374-8265.10963)
Supplement: Supplementary file 1 — CPAP Desensitization.pptxCPAP Interactive Role-Play.docxCPAP Desensitization Patient Protocol.docxCPAP Pre- & Posttest.docx [file mep_2374-8265.10963-s001.zip › _Educational_Summary_Report_10963.pdf]

MedEdPORTAL® The AAMC Journal of Teaching and Learning Resources

Original Publication Open Access

Improving CPAP Adherence for Obstructive Sleep Apnea: A Practical Application Primer on CPAP Desensitization

Yelena Chernyak, PhD*

*Corresponding author: ychernya@iupui.edu

Abstract

Introduction: Obstructive sleep apnea (OSA) is a common medical condition with well-established morbidity and mortality. Continuous positive airway pressure (CPAP) is a highly effective treatment prescribed to most individuals with OSA that has documented poor adherence rate for a variety of reasons including claustrophobia and discomfort. CPAP desensitization is an effective, simple, and brief treatment shown to improve adherence rates to CPAP. **Methods:** A psychologist specializing in behavioral sleep medicine developed this module focused on teaching medical residents the techniques of CPAP desensitization. The educational activity was an interactive 45-minute seminar which included a didactic component followed by a case presentation and interactive role-play. A postseminar survey was used to evaluate the content of the workshop, as well as growth in awareness and perception of knowledge and skills with a pre- to postworkshop evaluation. **Results:** In a survey of 25 primary care and psychiatry residents and sleep medicine fellows, 92% of respondents indicated that the topic of CPAP barriers and CPAP desensitization was important. Ratings of self-reported knowledge and skills improved nearly one-third following the workshop. Qualitative feedback indicated the utility and enthusiasm learners had for this topic. **Discussion:** The workshop on CPAP desensitization was a valuable tool that should be disseminated more widely to improve treatment adherence in the significant portion of the population that suffers from OSA which does not use adherence to positive airway pressure therapy. The workshop is applicable to other health professionals including medical students and nursing, social work, or psychology trainees.

Keywords
Sleep Apnea, Obstructive Sleep Apnea, CPAP, PAP Therapy, Sleep, Desensitization, Preventive Medicine, Primary Care, Psychiatry, Pulmonary Medicine, Sleep Medicine, Case-Based Learning, Clinical/Procedural Skills Training

Educational Objectives

By the end of this seminar, medical learners will be able to:

1. Identify three or more common barriers to compliance with continuous positive airway pressure (CPAP) therapy for sleep apnea.
2. List three or more components of CPAP desensitization intervention.
3. Identify three or more clinical characteristics of an appropriate candidate for CPAP desensitization referral and/or intervention.

Citation:
Chernyak Y. Improving CPAP adherence for obstructive sleep apnea: a practical application primer on CPAP desensitization. *MedEdPORTAL*. 2020;16:10963.
https://doi.org/10.15766/mep_2374-8265.10963

Introduction

Epidemiological studies indicate 26% of Americans are affected by obstructive sleep apnea (OSA), a condition resulting in adverse outcomes such as motor vehicle accidents, cardiac events, and poor quality of life leading to substantial morbidity and mortality.[1] The vast majority of individuals with OSA are prescribed continuous positive airway pressure (CPAP) therapy as the gold standard of treatment for reducing apneic events with well documented benefits to quality of life.[2-3]

Adherence remains the biggest obstacle in the implementation of CPAP therapy. The Medicare-defined minimum use threshold of 4 or more hours for 70% of nights had an estimated 46% adherence rate in early studies.[4] Advances in CPAP technology and comfort have not substantially improved adherence, now ranging from 29%-83% in recent studies.[5] Moreover, these adherence estimates are underrepresenting the problem which is, ideally, most patients need to far exceed the Medicare-defined

Copyright © 2020 Chernyak. This is an open-access publication distributed under the terms of the Creative Commons Attribution license.

minimum use for optimal outcomes and tolerate CPAP throughout entire sleep duration.

Leaders in the field of sleep medicine, including Crawford and Espie, suggest that psychological and medical treatment must be integrated in CPAP treatment to improve adherence rates.[6] Claustrophobia is the largest deterrent to CPAP therapy, cited by as many as 63%-84% of respondents in previous studies and associated with greater risk of poor CPAP adherence or abandonment.[7-8] It is characterized by a feeling of being uncomfortable with the CPAP mask, positive pressure, or both, causing anxiety, worry, avoidance, and, often, insomnia. CPAP desensitization is a type of graded exposure therapy to feared stimuli—namely CPAP related claustrophobia and discomfort.[9]

In combination with comfort measures such as selecting the most comfortable mask interface, humidifier, and nasal decongestant to use, CPAP desensitization is a safe and effective tool for addressing CPAP nonadherence and claustrophobia. Edinger and Radtke first published a case report in 1993 on CPAP desensitization,[10] while Means and Edinger went on to develop a CPAP-specific desensitization therapy protocol designed to be delivered in six sessions or less over the duration of 1-3 months.[11] Several case studies and case series have been published documenting large effect sizes, with gains of 2.6 hours per night and 2.4 nights per week in one such study.[12-13] Although large treatment outcome studies have not been conducted on CPAP desensitization specifically, exposure therapy and desensitization are exceedingly well researched behavioral therapies with a large literature base supporting their use in a variety of phobic conditions, including claustrophobia, as well as anxiety disorders.[14-16] Moreover, given the lack of other efficacious strategies for improving CPAP adherence, the benefit to patients of improved adherence, and the minimal costs and risks of implementing a behavioral intervention such as CPAP desensitization, there is little reason not to offer this treatment to patients more widely.

CPAP desensitization remains underutilized likely due to the lack of awareness of its efficacy and availability of qualified treatment providers. Therefore, training medical providers in this brief and effective strategy is important. Medical residents would benefit from increased training in CPAP desensitization to address the knowledge and skill gap in current de-facto medical education. A brief, implementable, workshop on CPAP desensitization that is appropriate for general medical trainees or practitioners would be an important addition to the medical education literature. This seminar should consist of a didactic component to fill the knowledge gap on OSA and CPAP nonadherence, given the dearth of formal education on these topics in generalist settings. The didactic component should be followed by an interactive case presentation and role-play vignettes to illustrate the application of knowledge with tools. Education highlighting the importance of CPAP compliance in the context of known barriers combined with an immediately applicable skillset to target those barriers will hopefully bridge the gap in utilization so many individuals with sleep apnea are experiencing. There is nothing currently available in the *MedEdPORTAL* on managing barriers to CPAP adherence in OSA or CPAP desensitization. This seminar was designed to fill a significant gap in medical training surrounding CPAP use.

Methods

This seminar was developed by an academic clinical health psychologist who is board certified in behavioral sleep medicine by the American Academy of Sleep Medicine. The purpose of this seminar was training advanced medical learners in CPAP desensitization at the residency or fellowship level. Basic knowledge about sleep apnea, CPAP, or behavioral therapy, generally, was not a prerequisite to benefit from this workshop. This workshop was facilitated during Indiana University School of Medicine behavioral sleep medicine rotations, including those for the family medicine and psychiatry residencies. The workshop length was 45 minutes.

A basic introduction to sleep apnea physiology and CPAP intervention was presented, summarizing basic information from the Academy of Sleep Medicine website.[17] The content was summarized in a PowerPoint presentation (Appendix A), which was projected on a large screen in a private meeting room. Copies of slides were provided to learners. One vignette was reviewed at the conclusion of the presentation that illustrated CPAP desensitization treatment response. Relevant cases were also elicited from the learners' active caseloads as illustrative examples. An optional interactive exercise was conducted to role-play training in CPAP desensitization (Appendix B) following the presentation. A summary sheet of the CPAP desensitization protocol for learners to keep was provided (Appendix C).

The audience consisted primarily of internal medicine residents, but also included psychiatry residents and sleep medicine fellows. Each workshop was presented with five or more learners. Learners were seated in a semicircle fashion to facilitate question and answer opportunities as well as discussion.

Assessment

Immediately prior to the workshop, learners completed a 10-item pretest on their knowledge, skills, and interest of the treatment

Copyright © 2020 Chernyak. This is an open-access publication distributed under the terms of the Creative Commons Attribution license.

topic (Appendix D). The same 10 items were readministered immediately following the workshop to assess perception of knowledge, skills, and interest gained via self-report. In addition, the posttest also included ratings of satisfaction and quality of the workshop. Ratings of each content area were evaluated on a Likert scale ranging from *strongly agree* to *strongly disagree*. Answers of *strongly agree*, *agree*, and *neutral* were considered favorable responses to the content areas evaluated. The last item asking for a global rating of the workshop was reverse scored, such that lower scores indicated more favorable response. Respondents could also leave qualitative comments or suggestions as part of the survey. Learners' participation in or responses to the survey was anonymous, voluntary, and did not affect evaluation of their performance by supervisors.

Results

Data were collected from 25 learners, 12 of whom were internal medicine residents, three of whom were sleep medicine fellows, four of whom were psychiatry residents, and six of whom were psychology residents at a large urban medical school. Seminars were conducted in small groups ranging from four to six participants. At the conclusion of the seminar all learners overwhelmingly felt that the educational objectives had been met.

Workshop Evaluation

The posttest indicated that all (100%) of the respondents felt this workshop was either *good* (12%) or *excellent* (88%). With regard to the format of the workshop, the agreement with the statements, "This workshop was well organized," "I found this small group format useful," "This format was valuable for learning this topic," and "The interactive component of this topic was useful" was high across the board with average ratings of 4.7, 4.4, 4.6, and 4.6 (based on a 5-point Likert scale), respectively. The audio-visual aids were rated as "reinforcing the content" with a favorable 4.7 average rating (Table 1).

Pre- to Posttest Perceived Knowledge and Skills Evaluation

The seminar evaluation form also included 10 assessment items which were administered as a pre- and posttest. Nearly all (92%) learners selected either *agree* or *strongly agree* when asked if this topic was important, with the average rating (4.4) remaining constant from pre- to posttest (Table 1).

Growth was demonstrated on survey items measuring perceived knowledge and skills related to the topic (Table 1). Most notably, the four items with the lowest pretest average ratings—"I feel comfortable evaluating/diagnosing on this topic" (2.2), "I have the skills to evaluate/diagnose on this topic" (2.2), "I feel comfortable solving clinical problems on this topic" (2.5), and "I have the clinical skills to solve clinical problems on this topic" (2.4)—showed the greatest gains following the workshop. Average rating increases ranged from 1.4 to 1.6 points on all four items with posttest average ratings of 3.7, 3.6, 4.0, and 4.0, respectively, on a 5-point Likert scale. The posttest average ratings for these four items most closely corresponded to the *agree* (4) statement of the Likert scale. Substantial improvement was also notable on the understanding the "clinical development of this topic" with a posttest average rating of 4.2, a 1.1-point increasing from pre- to posttest, as well as the understanding of the "illness surrounding the topic," with a posttest average rating of 4.1, a 0.7-point pre- to posttest increase. The workshop even slightly increased the already high level of enthusiasm for the topic with a final average rating of 4.4, which was a 0.3-point increase from pre- to posttest.

The lowest rated item on the evaluation was in response to the statement, "I will encounter many opportunities to use this clinical skill," with a rating of 3.3 the majority (64%) of respondents rated this as *neutral*, with no change pre-to posttest. However, ironically, the only other item that did not change from pre- to posttest was the highly rated, "I would like to seek out more opportunities to use this clinical skill," which had a rating of 4.4 where the majority (52%) of respondents rated this as *strongly agree* (Table 1).

Qualitatively, learners provided written feedback on the posttest survey. Sample comments included the following: "easy to understand," "straightforward," "informative to talk through …practicalities of actual case." Solicited recommendations for changes to the workshop included comments such as, "incorporate more cases, would like to learn more about integration with cognitive behavioral therapy for insomnia, nice to learn about …common mistakes [of CPAP use]." The author performed a thematic analysis of the qualitative comments from the workshops, which indicated strengths of the seminar included the didactic and applied nature of the presentation, while recommendations for improvement focused on more in-depth case review and integration of knowledge about CPAP equipment/masks as well as integration with insomnia treatment (Table 2). Overall, these qualitative responses indicated objectives were met to increase awareness and knowledge of barriers to CPAP compliance and improve skills to implement CPAP desensitization.

Discussion

This seminar was designed to provide an overview of barriers to CPAP compliance with an introduction to CPAP desensitization as

Copyright © 2020 Chernyak. This is an open-access publication distributed under the terms of the Creative Commons Attribution license.

Table 1. Continuous Positive Airway Pressure Adherence Evaluation Pre- & Posttest Responses (*N* = 25)

Rating Items	Strongly Disagree (%)	Disagree (%)	Neutral (%)	Agree (%)	Strongly Agree (%)	Average[a]	Change
1. This is an important topic for a clinical workshop.							
Pre			8	40	52	4.4	
Post			8	40	52	4.4	0
2. I understand clinical development of this topic.							
Pre	4	4	68	24		3.1	
Post			4	72	24	4.2	1.1
3. I understand the illness surrounding this topic.							
Pre			64	36		3.4	
Post			20	52	28	4.1	0.7
4. I feel comfortable evaluating/diagnosing on this topic.							
Pre	8	73	8	12		2.2	
Post			52	28	20	3.7	1.5
5. I have the skills to evaluate/diagnose on this topic.							
Pre	20	56	12	12		2.2	
Post		16	20	56	16	3.6	1.4
6. I feel comfortable solving clinical problems on this topic.							
Pre	4	56	24	16		2.5	
Post			24	56	20	4.0	1.5
7. I have the skills to solve clinical problems on this topic.							
Pre	4	64	16	16		2.4	
Post			20	60	20	4.0	1.6
8. I am enthusiastic about applying/practicing this clinical skill.							
Pre			8	72	20	4.1	
Post			8	64	44	4.4	0.3
9. I will encounter many opportunities to use this clinical skill.							
Pre		8	64	16	12	3.3	
Post		8	64	16	12	3.3	0
10. I would like to seek out more opportunities to use this clinical skill.							
Pre			8	40	52	4.4	
Post			8	40	52	4.4	0
11. This workshop was well organized.							
Post			4	20	76	4.7	
12. I found this small group format useful.							
Post		4	4	40	52	4.4	
13. The facilitators effectively conducted this exercise.							
Post			4	24	72	4.7	
14. This format was valuable for learning this topic.							
Post			8	28	64	4.6	
15. The facilitator appeared knowledgeable on this topic.							
Post					100	5.0	
16. The interactive component of this topic was useful.							
Post			12	12	76	4.6	
17. Audio/visual aids reinforced the content.							
Post			4	24	72	4.7	

[a]Rated on a 5-point Likert scale (1 = *strongly disagree*, 5 = *strongly agree*)

Table 2. Thematic Analysis of Feedback

Element of Presentation	Theme of Feedback	Student Comments
Strength	Brevity	"Easy to understand, straightforward."
	Applied nature	"Examples; [discussing] practicalities of actual case."
Recommended changes	Increase vignettes	"Incorporate more cases"
	In-depth learning	"Review common mistakes [of continuous positive airway pressure use]."
		"Learn about masks."
		"Integration with cognitive behavioral therapy for insomnia."

a brief and efficacious intervention to advanced medical learners. The didactic component was followed by an interactive case presentation and role-play vignettes, a combination of strategies intended to translate newly learned information about CPAP desensitization immediately into applied skill practice, in the hopes of breaching the divide between classroom learning and implementation. Although the didactic workshop is a common training technique in medical education, limitations may include difficulties with retention due to a rapid presentation of information, particularly in the absence of live cases to illustrate main points. This seminar was well received and

Copyright © 2020 Chernyak. This is an open-access publication distributed under the terms of the Creative Commons Attribution license.

achieved its objectives, demonstrated by overwhelmingly positive evaluations. The significant improvements in perceived knowledge, skills, and interest from pre- to postworkshop testing indicated that a substantial improvement in these areas can be delivered in a very brief interactive workshop. This very brief workshop was successful in improving self-reported knowledge and skills in the area of CPAP desensitization by nearly one-third. It is possible that for interested learners or specialized training settings, further brief education sessions may advance perceived skills and confidence further. Although this was designed primarily for resident physicians, it can be utilized with a variety of health professionals including medical students, nurses, and psychology trainees. Additionally, the seminar does not require prior knowledge of psychology, behavior therapy, or sleep disorders, making it accessible to a wide audience.

Interestingly, learners rated their interest in seeking out opportunities to practice related skills much higher than the likelihood of encountering opportunities to practice the skills by default. This may be indicative of a learning and training gap in medical education that does not arm trainees with the skills to evaluate and address barriers to CPAP noncompliance in standard practice that they feel would be utilized and would like to obtain. This discrepancy represented an important lesson learned in the implementation of this workshop in that learners may also need instruction on how to advocate for and disseminate the practice of CPAP desensitization in their professional settings so that it does become standard practice amongst their peers as well as future learners.

The main audio-visual supports used for this workshop included a brief PowerPoint presentation with an overview of positive airway pressure therapy, barriers, and a CPAP desensitization vignette. To enhance the likelihood that learners would feel confident implementing these techniques in clinical practice, a simple summary handout for learners was provided at the end of the session. This was also intended to serve as a jumping-off point for introducing this concept to patients quickly and efficiently in clinic. Lastly, educators were provided with an interactive role-playing exercise for practicing desensitization techniques in the small-group setting. Larger audiences of learners may not be as conducive to conducting the role-playing exercise presented. Due to the short nature of the workshop, interactive components such as the question and answer portion and the vignettes had to be limited due to time constraints. The time constraints experienced by medical learners as well as preceptors in fast-paced medical settings were challenging in the implementation of this workshop. Coordination amongst clinical training directors, preceptors, and clinical staff was critical in creating a training opportunity that would permit sufficient time to deliver this workshop. In real world practice, extending practice into case examples and role playing may further enhance the interactive component, which was one of the most highly rated elements of this workshop. As the importance of training on CPAP compliance and desensitization is disseminated and more widely accepted in medical settings, prioritizing this type of training amongst the competing demands of medical education may follow.

A strength of this workshop was the 100% evaluation completion rate. This goal was accomplished by scheduling time to complete both the pre-and postworkshop evaluations within the workshop itself, so that learners' competing demands were not limiting respondents' ability to complete the evaluation. The evaluation of learning was limited by the self-report nature of the feedback. Although the administration of both pre-and postworkshop questionnaires allowed us to ascertain change in awareness, perception of knowledge and skills, as well as confidence related to the topic of CPAP desensitization, actual change in knowledge and skills was not evaluated.

One final challenge to CPAP desensitization interventions was the lack of rigorous treatment outcome studies which might lend themselves to dissemination of treatment findings. However, despite the lack of significant funding of trials in the study of CPAP desensitization, it has a solid theoretical and practical basis which should be increasingly respected by medical professionals. The level of expertise to implement this technique is low such that this can be implemented successfully across the spectrum of health care providers. However, without further training, most medical providers will remain unaware this is a treatment option for CPAP non-adherence or claustrophobia. Delivering this primer about CPAP desensitization during medical education could help increase interest in this topic area and encourage further study, dissemination, and advocacy for this intervention to be delivered to the millions of Americans who are diagnosed with OSA and not obtaining maximal benefit from their positive airway pressure device.

Appendices

A. CPAP Desensitization.pptx

B. CPAP Interactive Role-Play.docx

Copyright © 2020 Chernyak. This is an open-access publication distributed under the terms of the Creative Commons Attribution license.

C. CPAP Desensitization Patient Protocol.docx

D. CPAP Pre- & Posttest.docx

All appendices are peer reviewed as integral parts of the Original Publication.

Yelena Chernyak, PhD: Assistant Professor, Department of Psychiatry, Indiana University School of Medicine; ORCID: https://orcid.org/0000-0001-6925-1981

Disclosures
None to report.

Funding/Support
None to report.

Ethical Approval
Reported as not applicable.

Reference

1. Peppard PE, Young T, Barnet JH, Palta M, Hagen EW, Hla KM. Increased prevalence of sleep-disordered breathing in adults. *Am J Epidemiol*. 2013;177(9):1006-1014. https://doi.org/10.1093/aje/kws342
2. Engleman HM, Asggari-Jirandeh N, McLeod AL, Ramsay CF, Deary IJ, Douglas NJ. Self-reported use of CPAP and benefit of CPAP therapy. *Chest*. 1996;109(6):1470-1476. https://doi.org/10.1378/chest.109.6.1470
3. Campos-Rodriquez F, Pena-Grinan N, Reyes-Nunez N, et al. Mortality in obstructive sleep apnea-hypopnea patients treated with positive airway pressure. *Chest*. 2005;128(2):624-633. https://doi.org/10.1378/chest.128.2.624
4. Kribbs NB, Pack AI, Kline LR, et al. Objective measurement of patterns of nasal CPAP use by patients with obstructive sleep apnea. *Am Rev Respir Dis*. 1993;147(4):887-895. https://doi.org/10.1164/ajrccm/147.4.887
5. Pruitt B. Top 10 Practices to Increase CPAP Compliance. *RT*. February 4, 2009. Accessed July 23, 2020. https://www.rtmagazine.com/products-treatment/top-10-practices-to-increase-cpap-compliance/
6. Crawford MR, Espie CA, Bartlett CJ, Grunstein RR. Integrating psychology and medicine in CPAP adherence—new concepts? *Sleep Med Rev*. 2014;18(2):123-139. https://doi.org/10.1016/j.smrv.2013.03.002
7. Edmonds JC, Yang H, King TS, Sawyer DA, Rizzo A, Sawyer AM. Claustrophobic tendencies and continuous positive airway pressure therapy non-adherence in adults with obstructive sleep apnea. *Heart Lung*. 2015;44(2):100-106. https://doi.org/10.1016/j.hrtlng.2015.01.002
8. Sawyer AM, Gooneratne NS, Marcus CL, Ofer D, Richards KC, Weaver TE. A systematic review of CPAP adherence across age groups: clinical and empiric insights for developing CPAP adherence interventions. *Sleep Med Rev*. 2011;15(6):343-356. https://doi.org/10.1016/j.smrv.2011.01.003
9. Leggett MK. A brief review of claustrophobia and continuous positive airway pressure (CPAP) therapy for sleep apnea. *J Sleep Med Disord*. 2016;3(2):1043.
10. Edinger JD, Radtke RA. Use of in vivo desensitization to treat a patient's claustrophobic response to nasal CPAP. *Sleep*. 1993;16(7):678-680.
11. Means MK, Edinger JD. Exposure therapy for claustrophobic reactions to continuous positive airway pressure. In: Perlis ML, Aloia M, Kuhn BR, eds. *Behavioral Treatments for Sleep Disorders: A Volume* in *Practical Resources for the Mental Health Professional*. 2011;183-193. https://doi.org/10.1016/B978-0-12-381522-4.00019-5
12. McCrae CS, Ingmundson PT. Using graduated in vivo exposure to treat a claustrophobic response to nasal continuous positive airway pressure: Hispanic male veteran associates nasal mask with gas masks worn during combat. *Clin Case Studies*. 2006;5(1):71-82. https://doi.org/10.1177/1534650103261203
13. Means MK, Edinger JD. Graded exposure therapy for addressing claustrophobic reactions to continuous positive airway pressure: a case series report. *Behav Sleep Med*. 2007;5(2):105-116. https://doi.org/10.1080/15402000701190572
14. Rachman S, Taylor S. Analyses of claustrophobia. *J Anxiety Disord*. 1993;7(4):281-291. https://doi.org/10.1016/0887-6185(93)90025-G
15. Wolitzky-Taylor KB, Horowitz JD, Powers MB, Telch MJ. Psychological approaches in the treatment of specific phobias: a meta-analysis. *Clin Psychol Rev*. 2008;28(6):1021-1037. https://doi.org/10.1016/j.cpr.2008.02.007
16. Choy Y, Fyer AJ, Lipsitz JD. Treatment of specific phobia in adults. *Clin Psychol Rev*. 2007;27(3):266-286. https://doi.org/10.1016/j.cpr.2006.10.002
17. American Academy of Sleep Medicine. Updated March 16, 2020. Accessed July 23, 2020. https://aasm.org/

Received: September 25, 2019
Accepted: February 26, 2020
Published: September 15, 2020

Copyright © 2020 Chernyak. This is an open-access publication distributed under the terms of the Creative Commons Attribution license.
