# Supplementary material for: Improving CPAP Adherence for Obstructive Sleep Apnea: A Practical Application Primer on CPAP Desensitization
Source: MedEdPORTAL. 2020 Sep 15;16:10963. doi: 10.15766/mep_2374-8265.10963 (PMC7499811; doi:10.15766/mep_2374-8265.10963)
Supplement: Supplementary file 1 — CPAP Desensitization.pptxCPAP Interactive Role-Play.docxCPAP Desensitization Patient Protocol.docxCPAP Pre- & Posttest.docx [file mep_2374-8265.10963-s001.zip › A. CPAP Desensitization.pptx]

## Slide 1
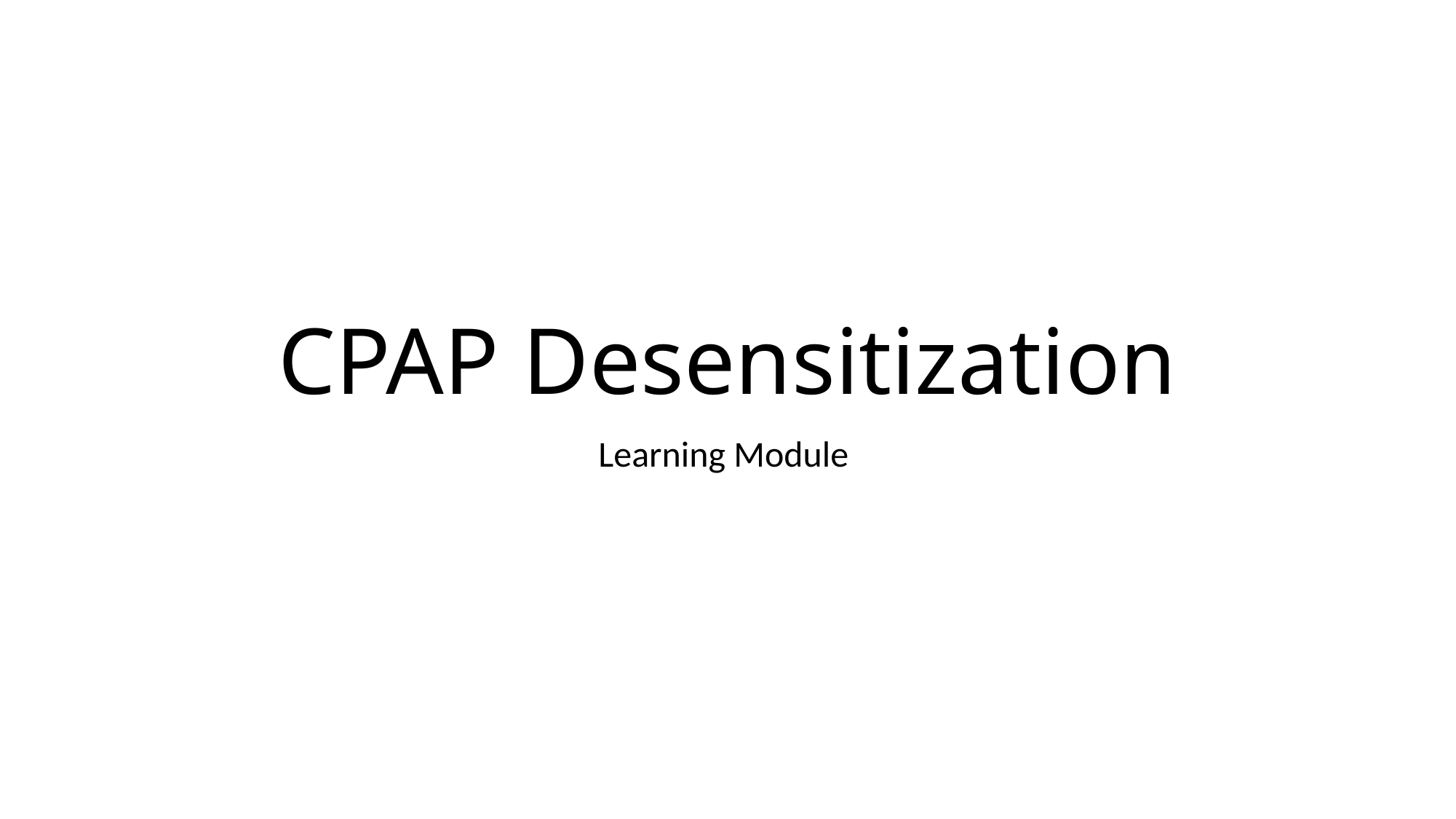

# CPAP Desensitization
Learning Module

## Slide 2
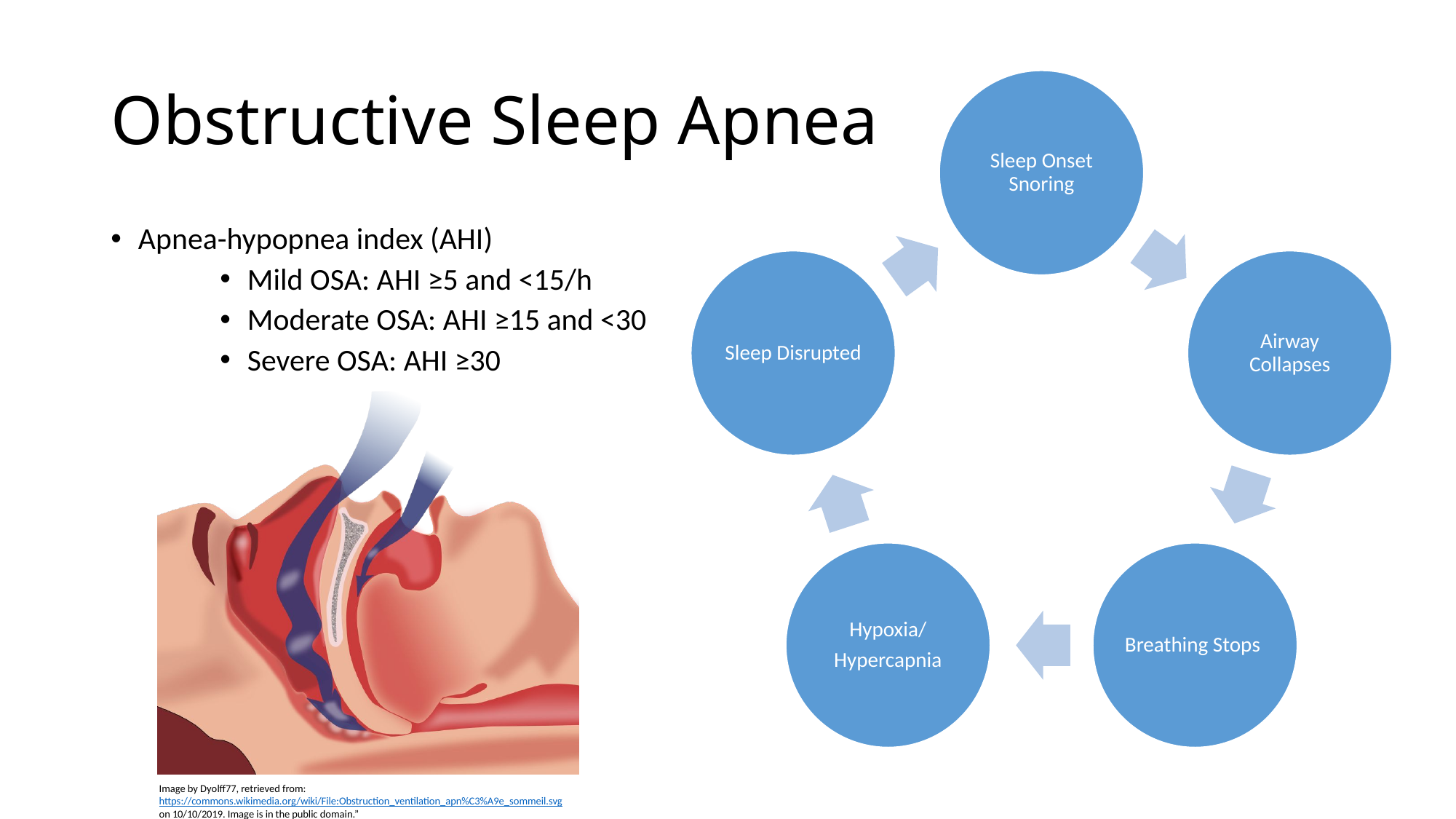

# Obstructive Sleep Apnea
Apnea-hypopnea index (AHI)
Mild OSA: AHI ≥5 and <15/h
Moderate OSA: AHI ≥15 and <30
Severe OSA: AHI ≥30
Image by Dyolff77, retrieved from: https://commons.wikimedia.org/wiki/File:Obstruction_ventilation_apn%C3%A9e_sommeil.svg on 10/10/2019. Image is in the public domain.”

## Slide 3
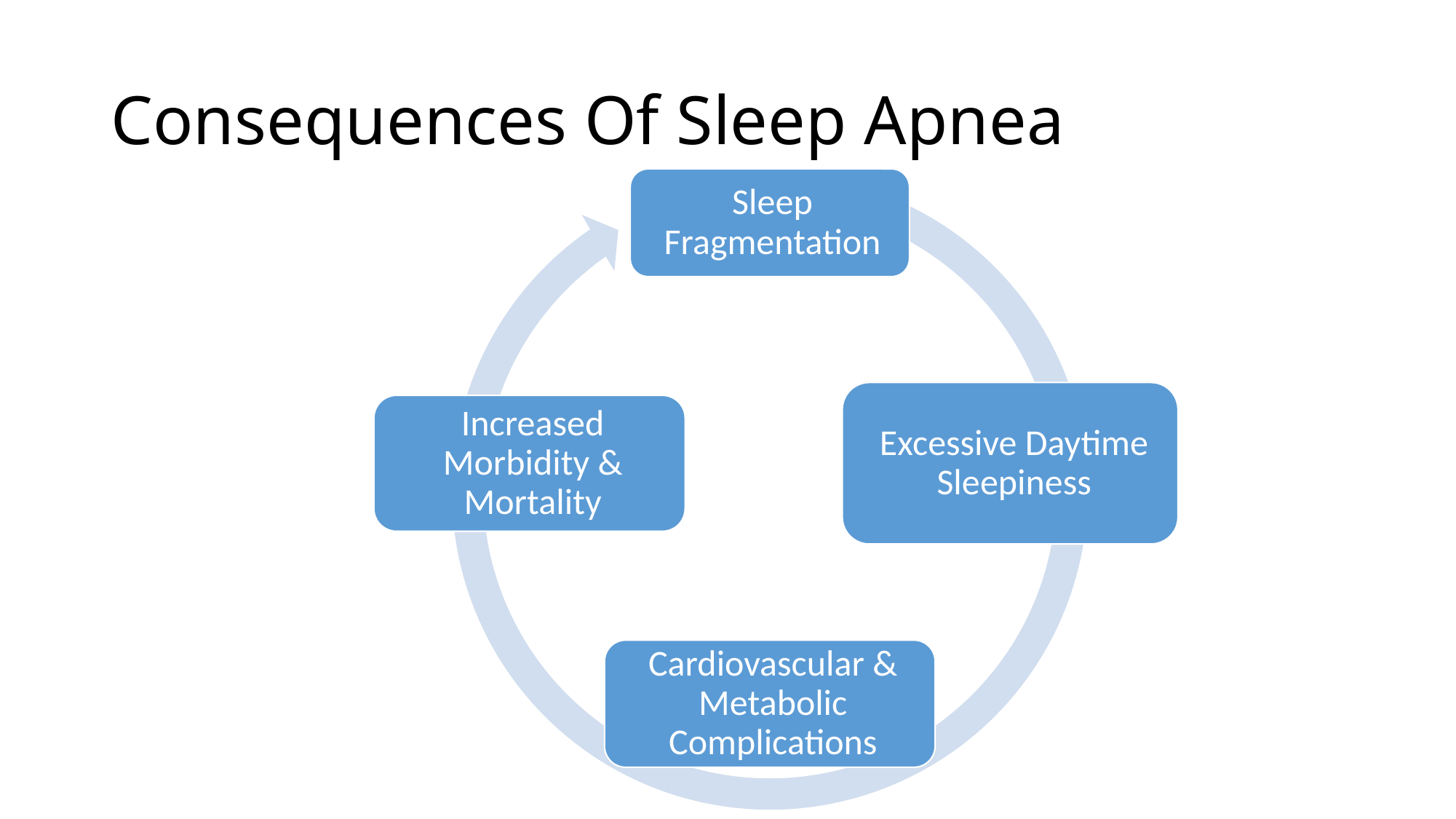

# Consequences Of Sleep Apnea

## Slide 4
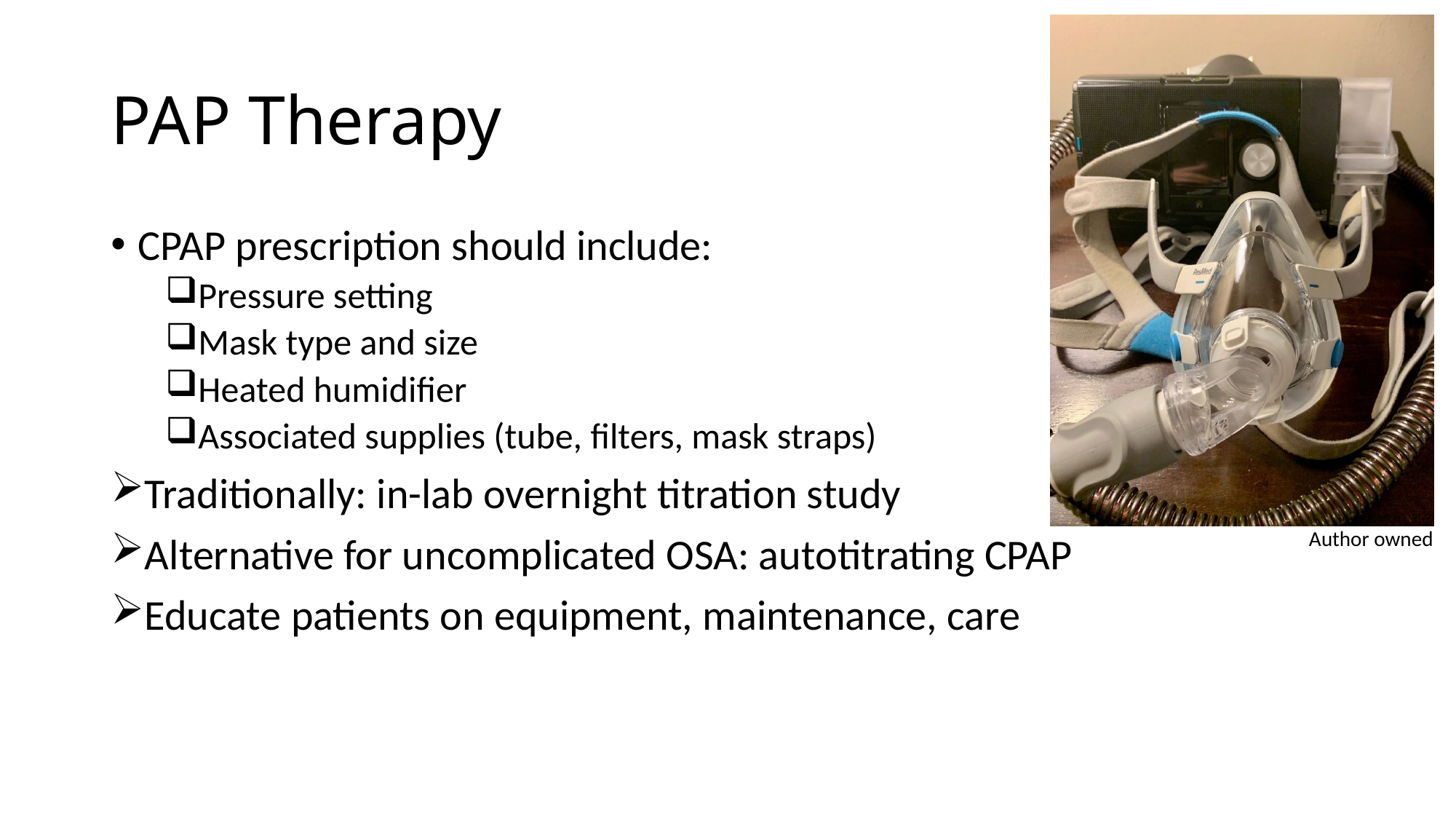

# PAP Therapy
CPAP prescription should include:
Pressure setting
Mask type and size
Heated humidifier
Associated supplies (tube, filters, mask straps)
Traditionally: in-lab overnight titration study
Alternative for uncomplicated OSA: autotitrating CPAP
Educate patients on equipment, maintenance, care
Author owned

## Slide 5
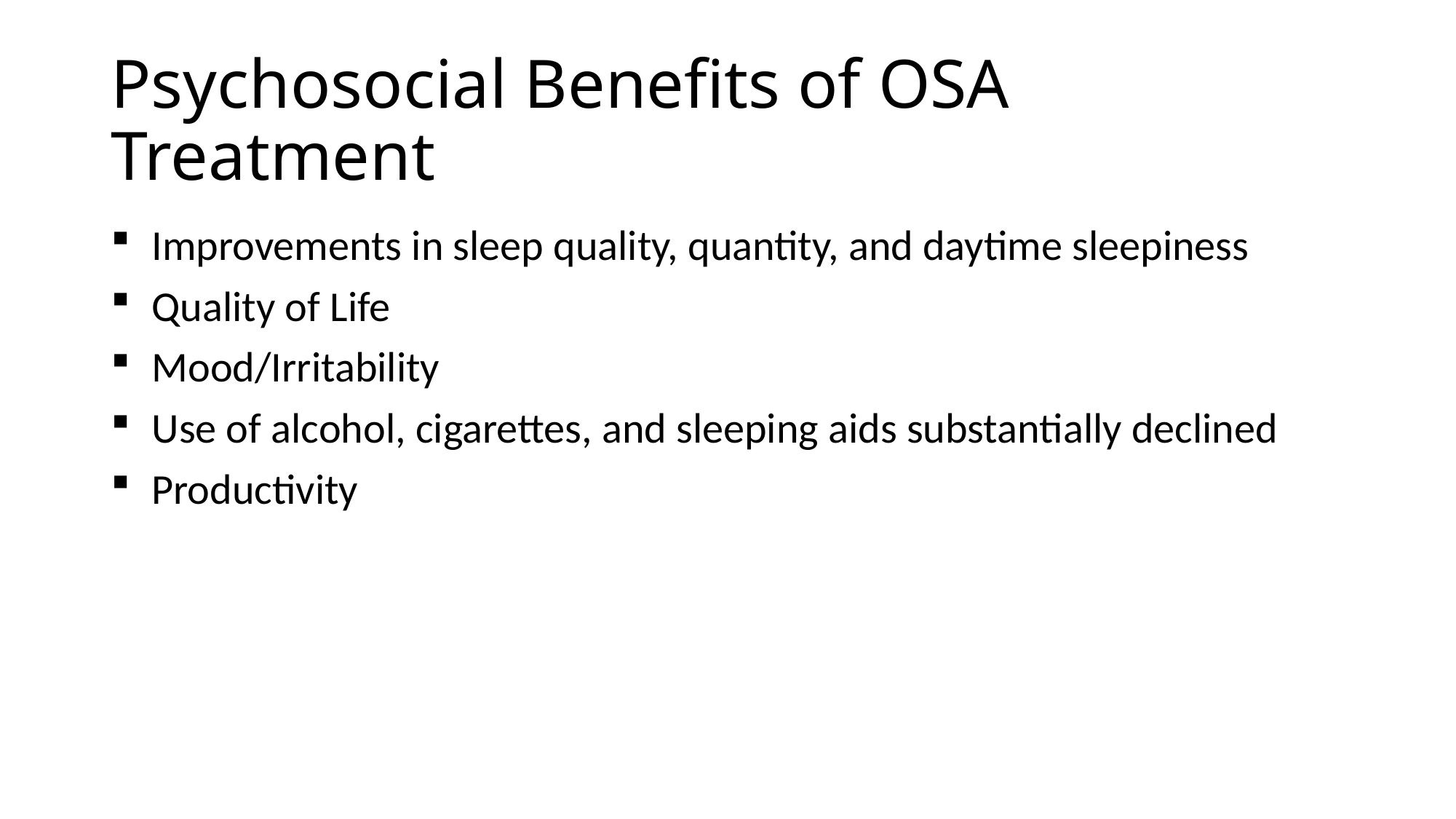

# Psychosocial Benefits of OSA Treatment
Improvements in sleep quality, quantity, and daytime sleepiness
Quality of Life
Mood/Irritability
Use of alcohol, cigarettes, and sleeping aids substantially declined
Productivity

## Slide 6
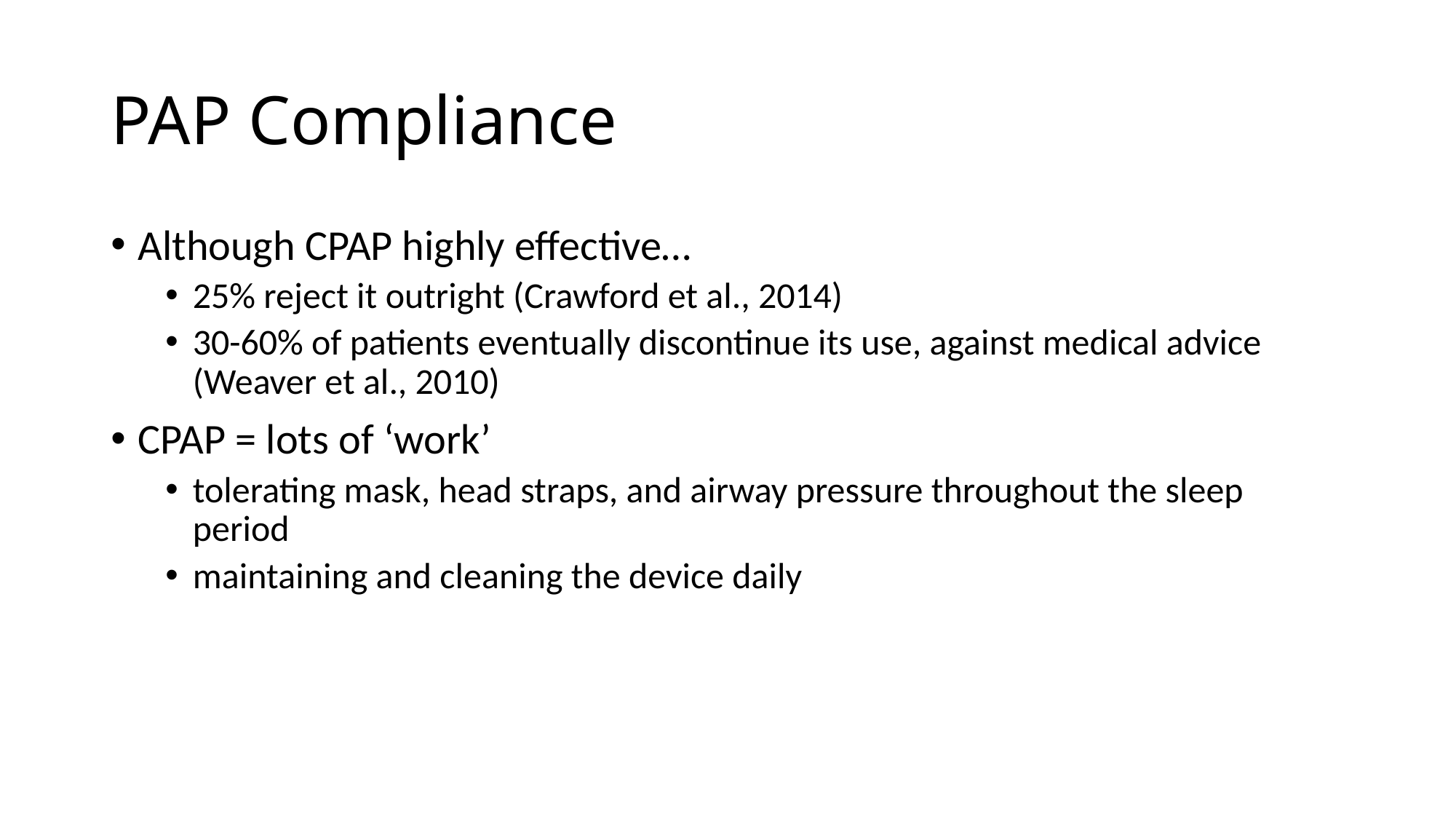

# PAP Compliance
Although CPAP highly effective…
25% reject it outright (Crawford et al., 2014)
30-60% of patients eventually discontinue its use, against medical advice (Weaver et al., 2010)
CPAP = lots of ‘work’
tolerating mask, head straps, and airway pressure throughout the sleep period
maintaining and cleaning the device daily

## Slide 7
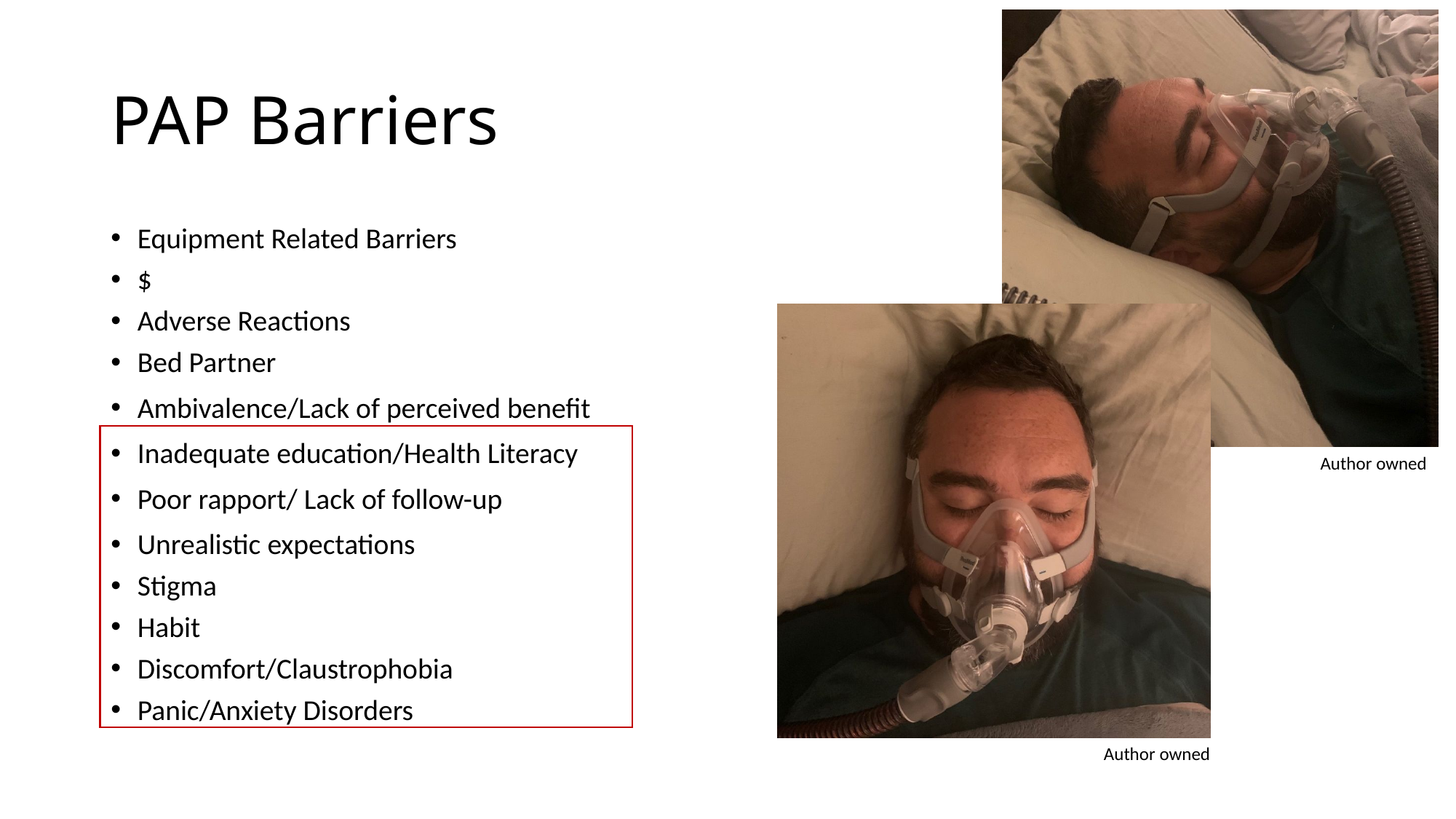

# PAP Barriers
Equipment Related Barriers
$
Adverse Reactions
Bed Partner
Ambivalence/Lack of perceived benefit
Inadequate education/Health Literacy
Poor rapport/ Lack of follow-up
Unrealistic expectations
Stigma
Habit
Discomfort/Claustrophobia
Panic/Anxiety Disorders
Author owned
Author owned

## Slide 8
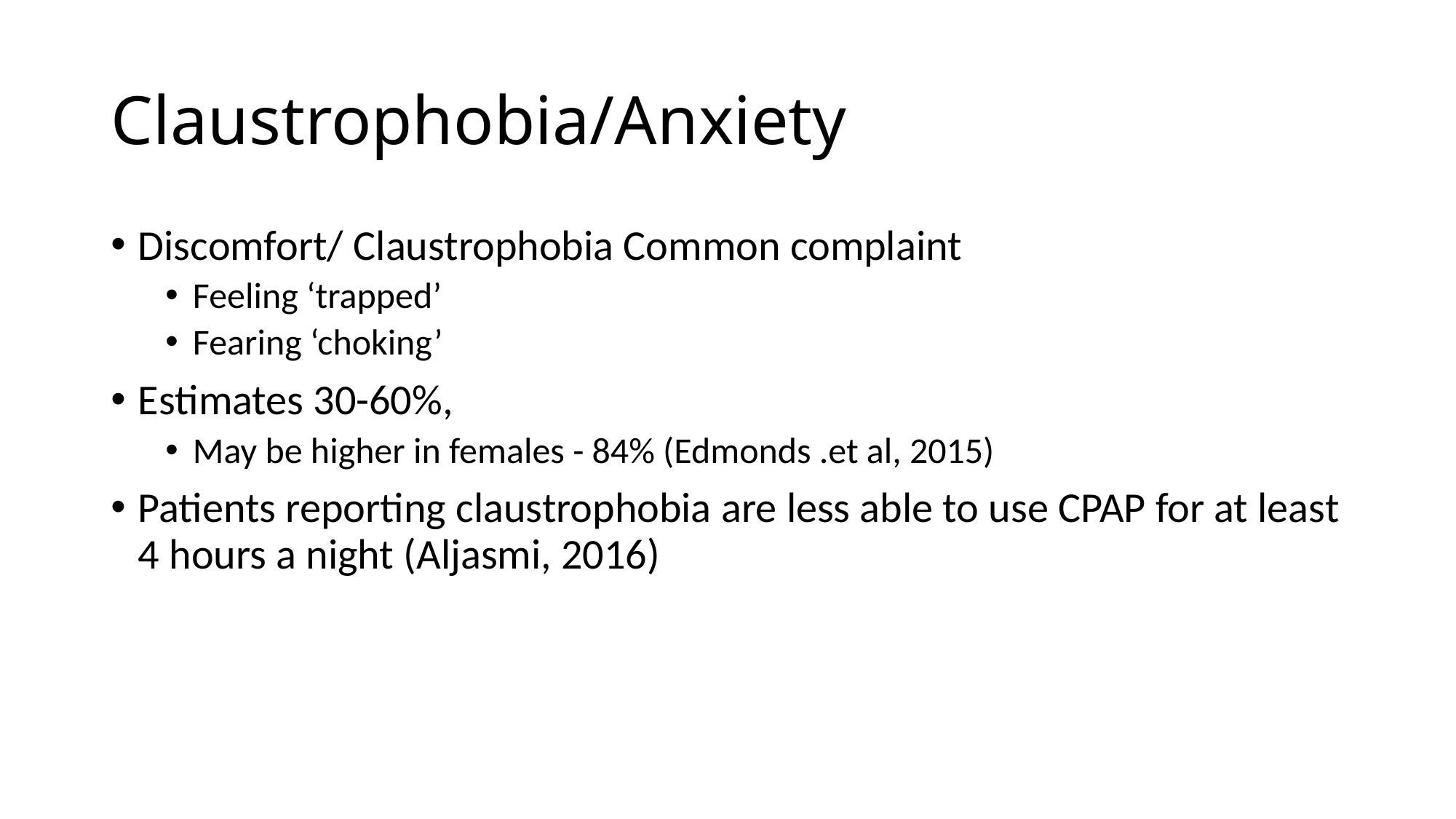

# Claustrophobia/Anxiety
Discomfort/ Claustrophobia Common complaint
Feeling ‘trapped’
Fearing ‘choking’
Estimates 30-60%,
May be higher in females - 84% (Edmonds .et al, 2015)
Patients reporting claustrophobia are less able to use CPAP for at least 4 hours a night (Aljasmi, 2016)

## Slide 9
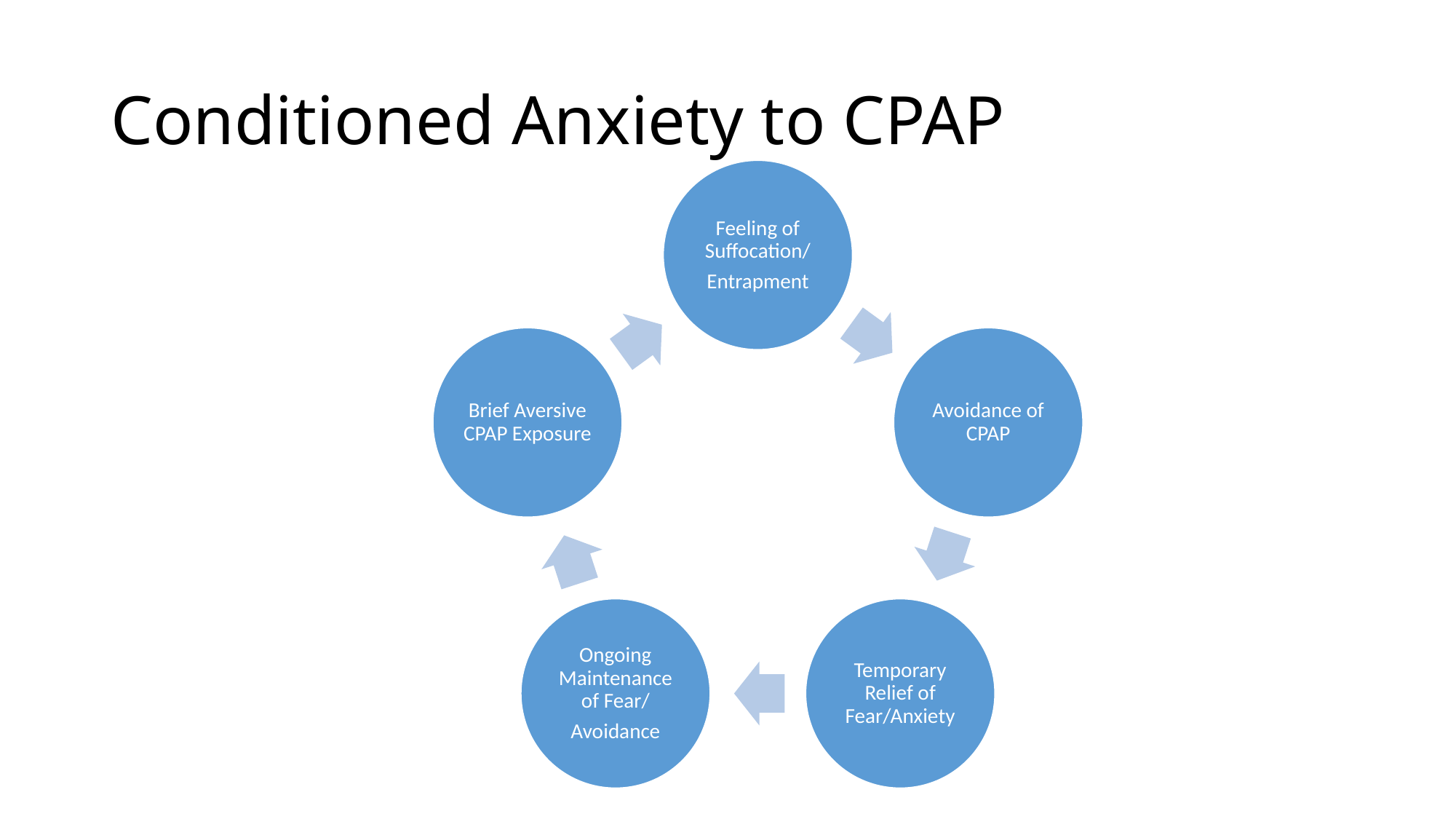

# Conditioned Anxiety to CPAP

## Slide 10
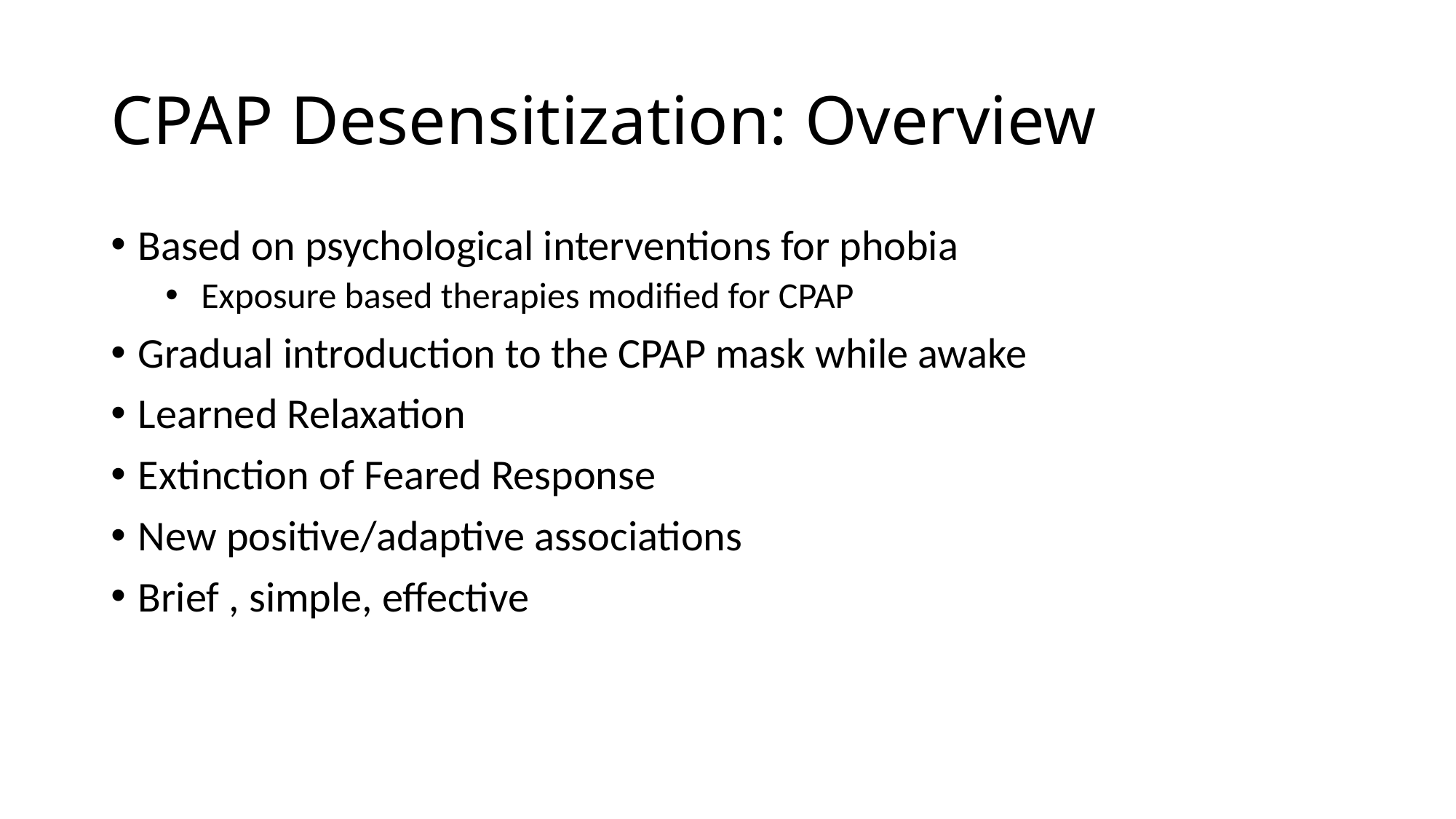

# CPAP Desensitization: Overview
Based on psychological interventions for phobia
 Exposure based therapies modified for CPAP
Gradual introduction to the CPAP mask while awake
Learned Relaxation
Extinction of Feared Response
New positive/adaptive associations
Brief , simple, effective

## Slide 11
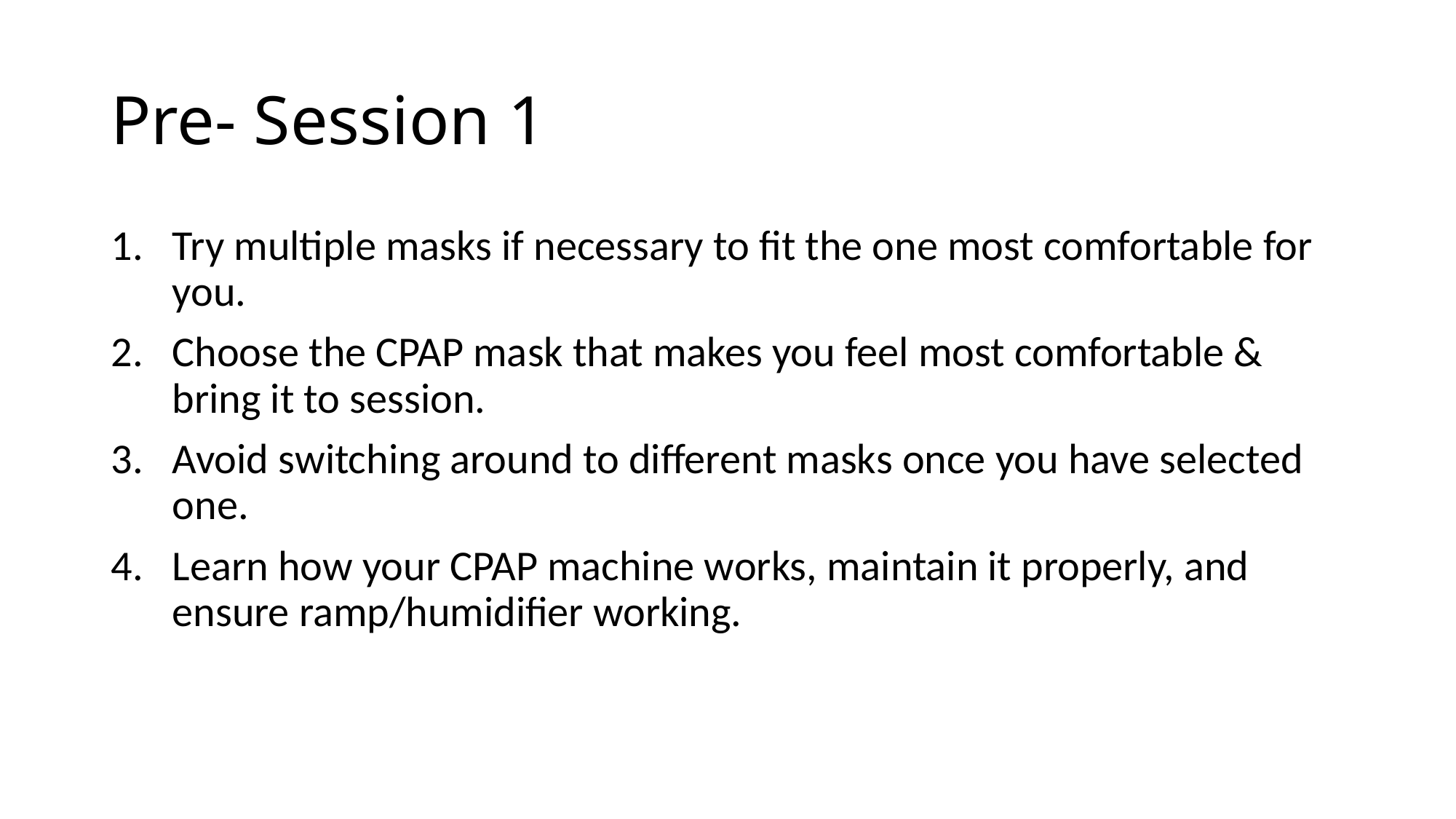

# Pre- Session 1
Try multiple masks if necessary to fit the one most comfortable for you.
Choose the CPAP mask that makes you feel most comfortable & bring it to session.
Avoid switching around to different masks once you have selected one.
Learn how your CPAP machine works, maintain it properly, and ensure ramp/humidifier working.

## Slide 12
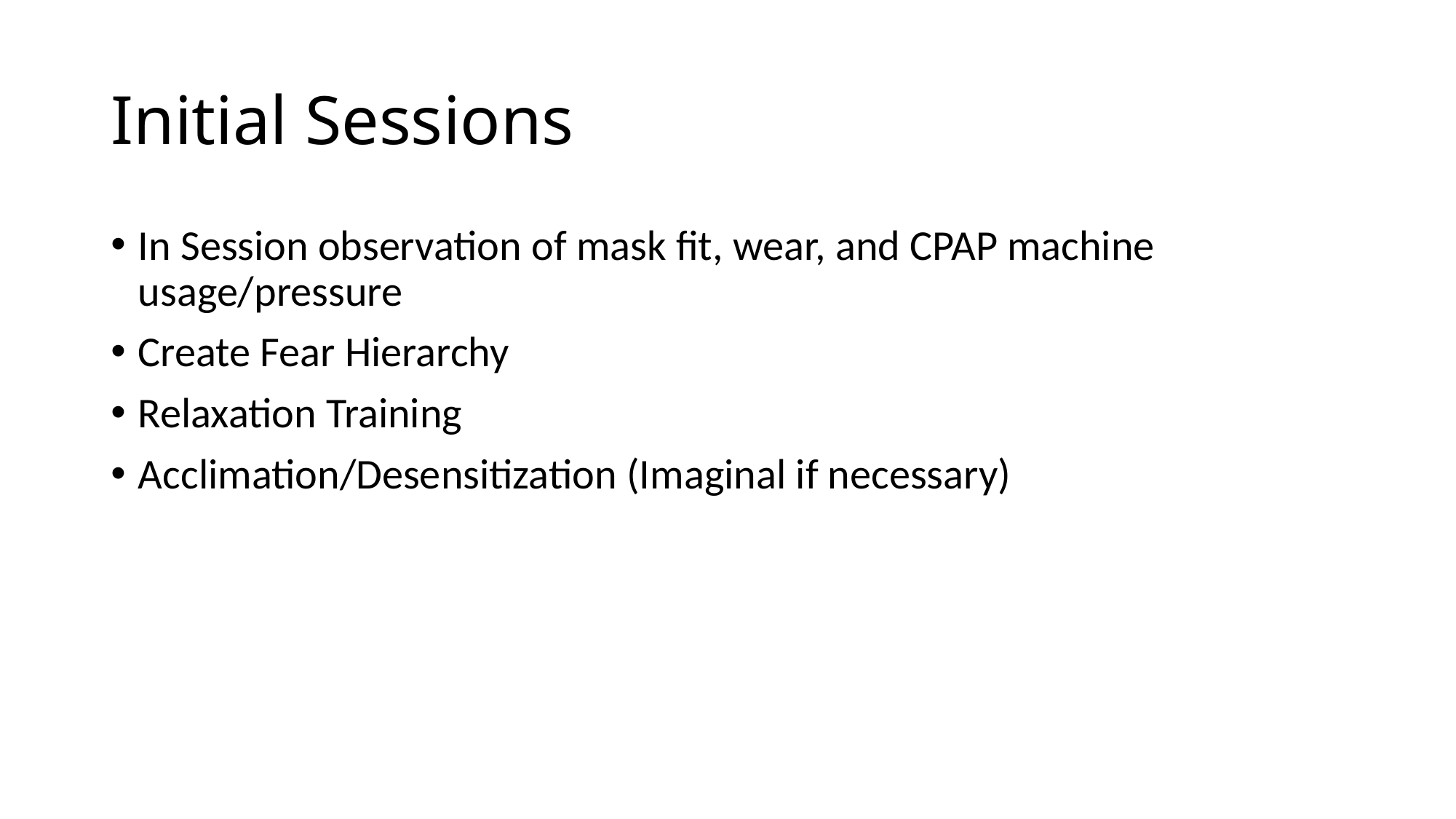

# Initial Sessions
In Session observation of mask fit, wear, and CPAP machine usage/pressure
Create Fear Hierarchy
Relaxation Training
Acclimation/Desensitization (Imaginal if necessary)

## Slide 13
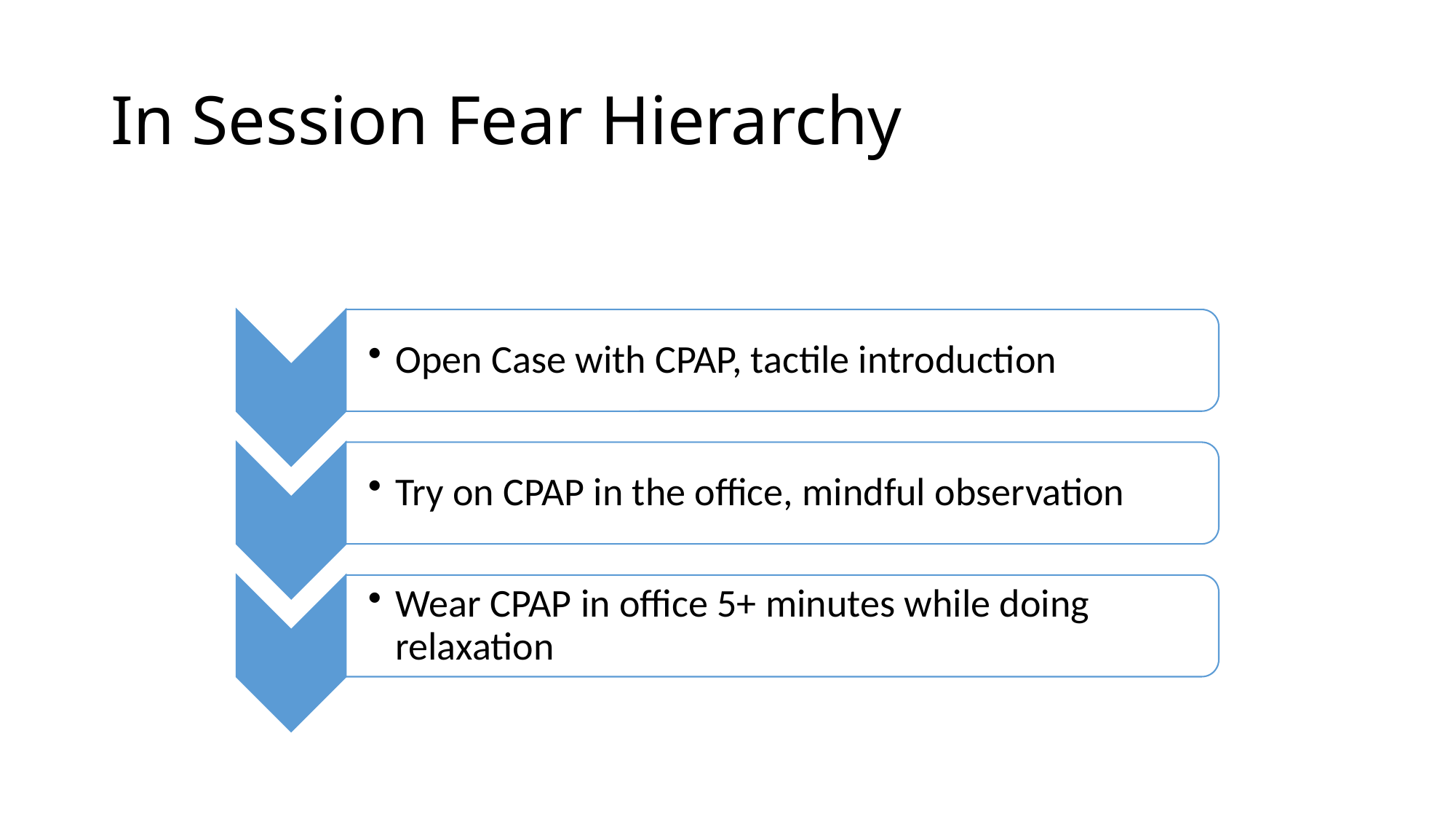

# In Session Fear Hierarchy

## Slide 14
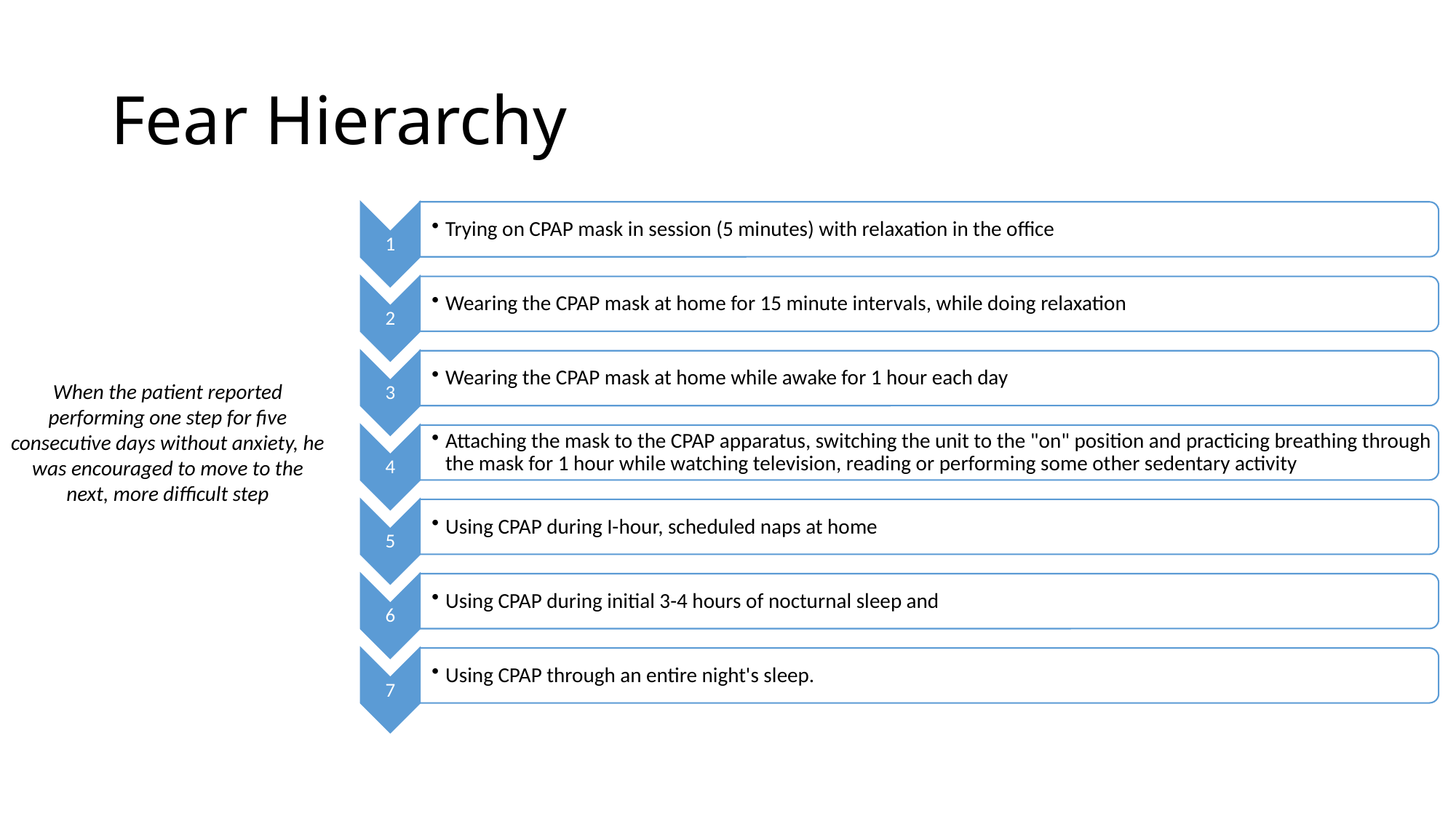

# Fear Hierarchy
When the patient reported performing one step for five consecutive days without anxiety, he was encouraged to move to the next, more difficult step

## Slide 15
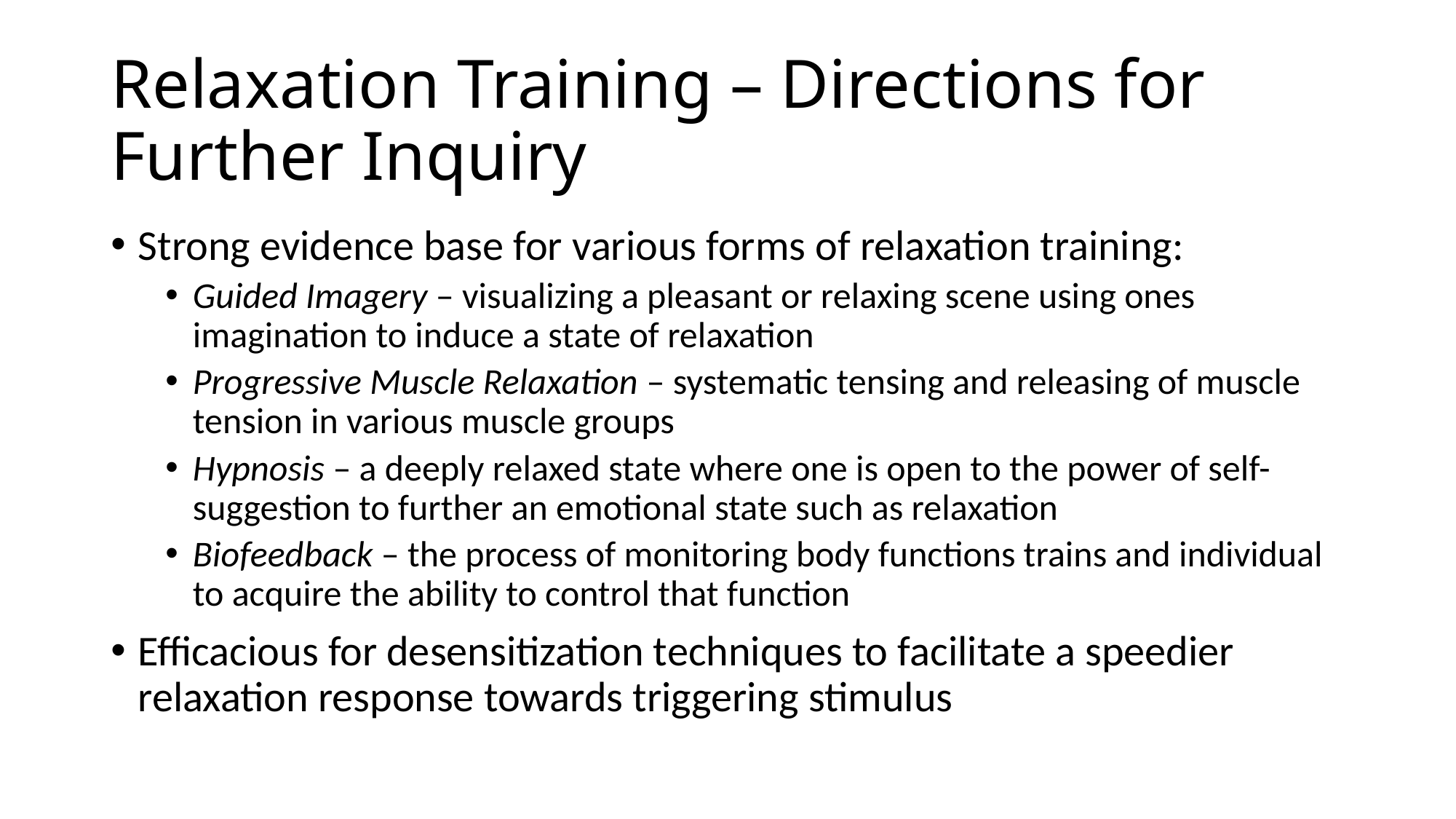

# Relaxation Training – Directions for Further Inquiry
Strong evidence base for various forms of relaxation training:
Guided Imagery – visualizing a pleasant or relaxing scene using ones imagination to induce a state of relaxation
Progressive Muscle Relaxation – systematic tensing and releasing of muscle tension in various muscle groups
Hypnosis – a deeply relaxed state where one is open to the power of self-suggestion to further an emotional state such as relaxation
Biofeedback – the process of monitoring body functions trains and individual to acquire the ability to control that function
Efficacious for desensitization techniques to facilitate a speedier relaxation response towards triggering stimulus

## Slide 16
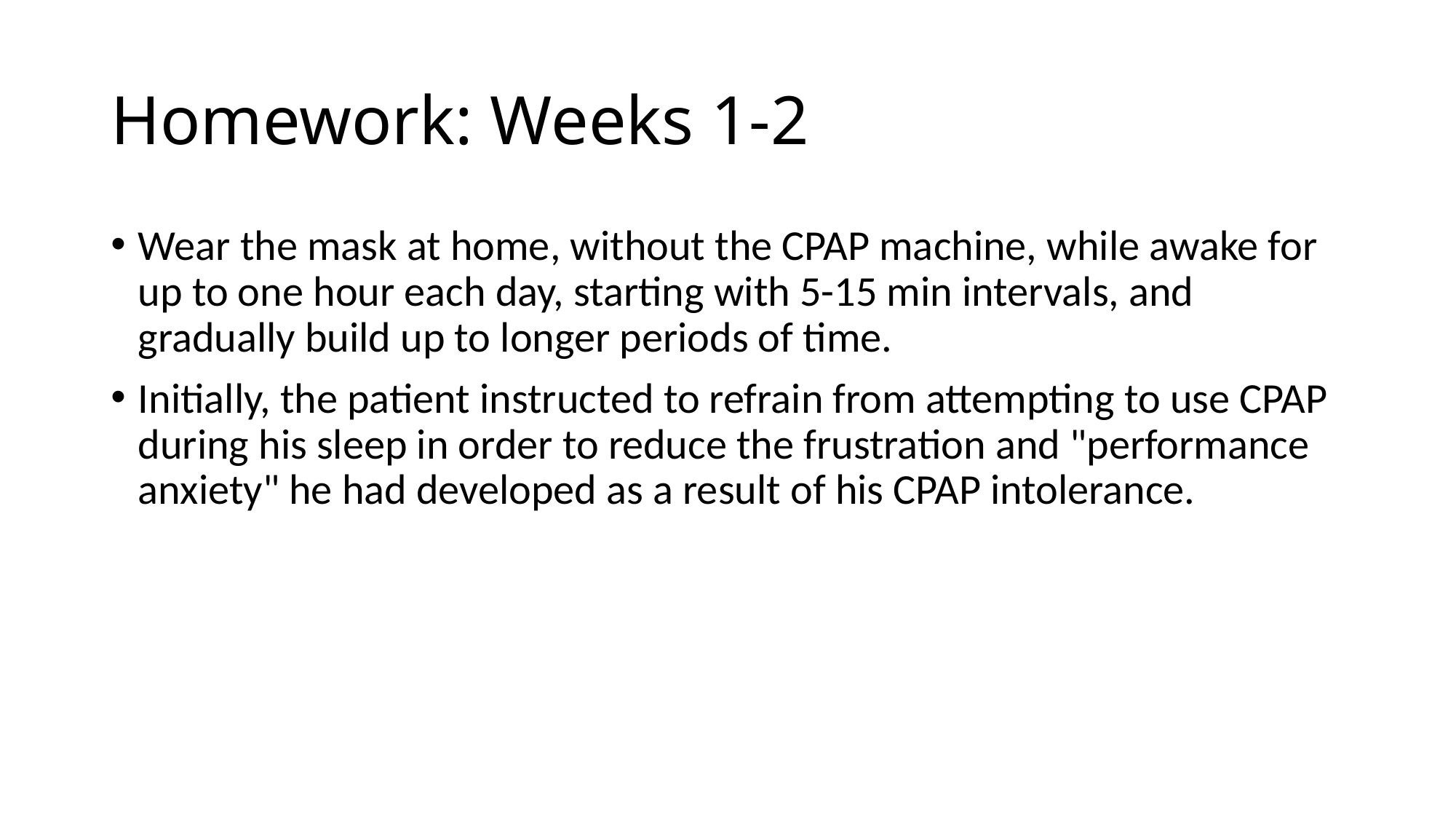

# Homework: Weeks 1-2
Wear the mask at home, without the CPAP machine, while awake for up to one hour each day, starting with 5-15 min intervals, and gradually build up to longer periods of time.
Initially, the patient instructed to refrain from attempting to use CPAP during his sleep in order to reduce the frustration and "performance anxiety" he had developed as a result of his CPAP intolerance.

## Slide 17
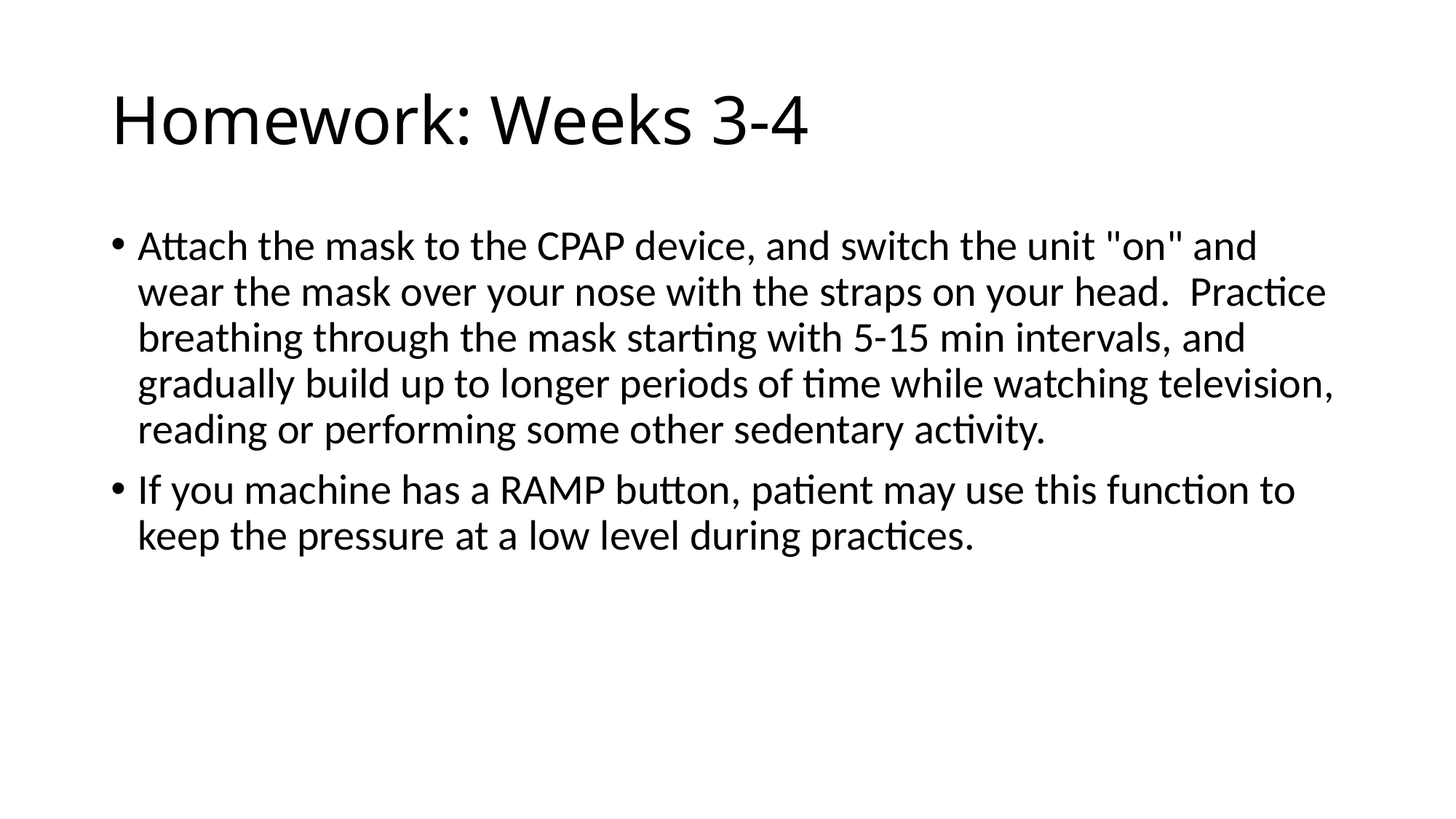

# Homework: Weeks 3-4
Attach the mask to the CPAP device, and switch the unit "on" and wear the mask over your nose with the straps on your head. Practice breathing through the mask starting with 5-15 min intervals, and gradually build up to longer periods of time while watching television, reading or performing some other sedentary activity.
If you machine has a RAMP button, patient may use this function to keep the pressure at a low level during practices.

## Slide 18
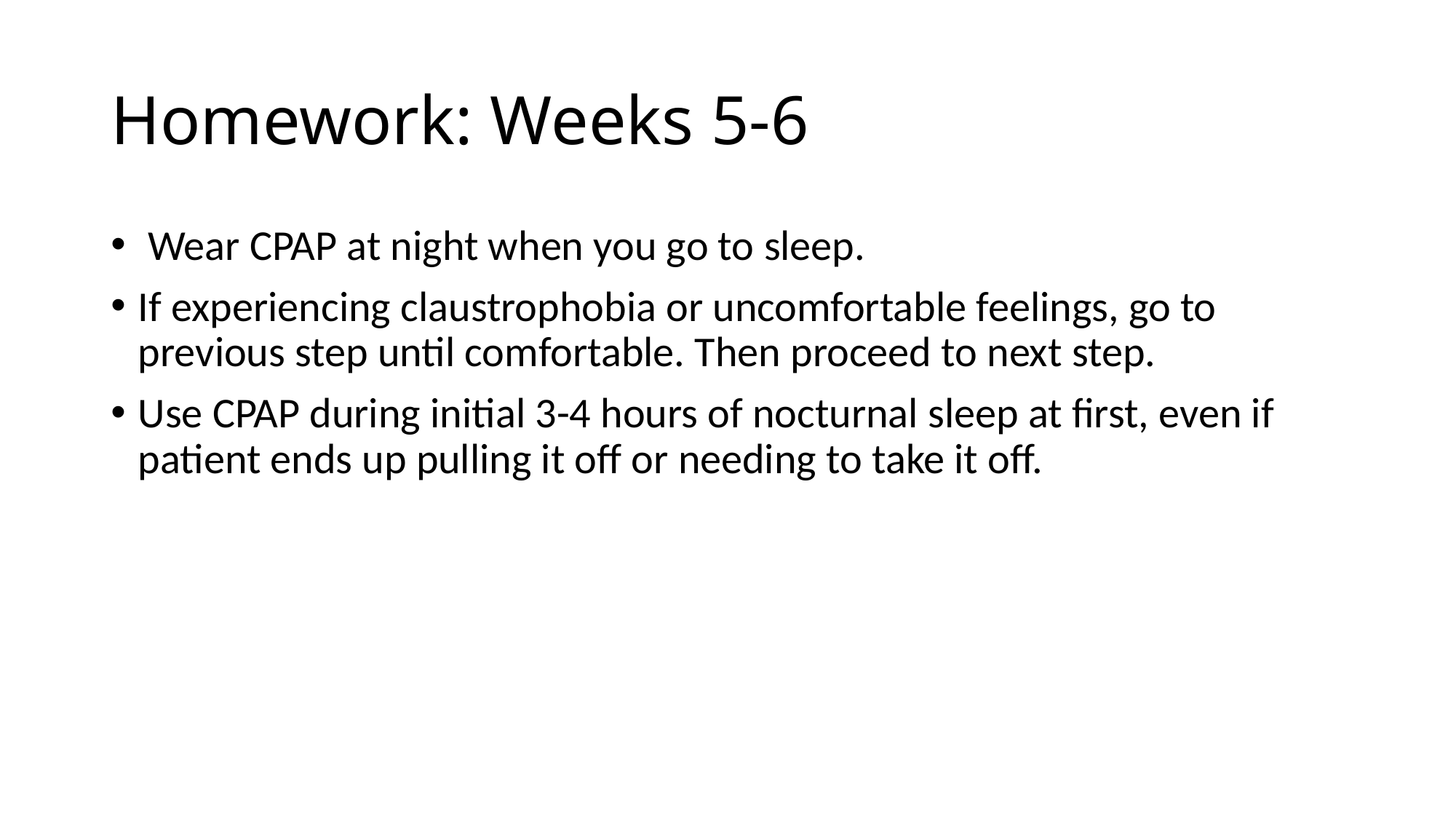

# Homework: Weeks 5-6
 Wear CPAP at night when you go to sleep.
If experiencing claustrophobia or uncomfortable feelings, go to previous step until comfortable. Then proceed to next step.
Use CPAP during initial 3-4 hours of nocturnal sleep at first, even if patient ends up pulling it off or needing to take it off.

## Slide 19
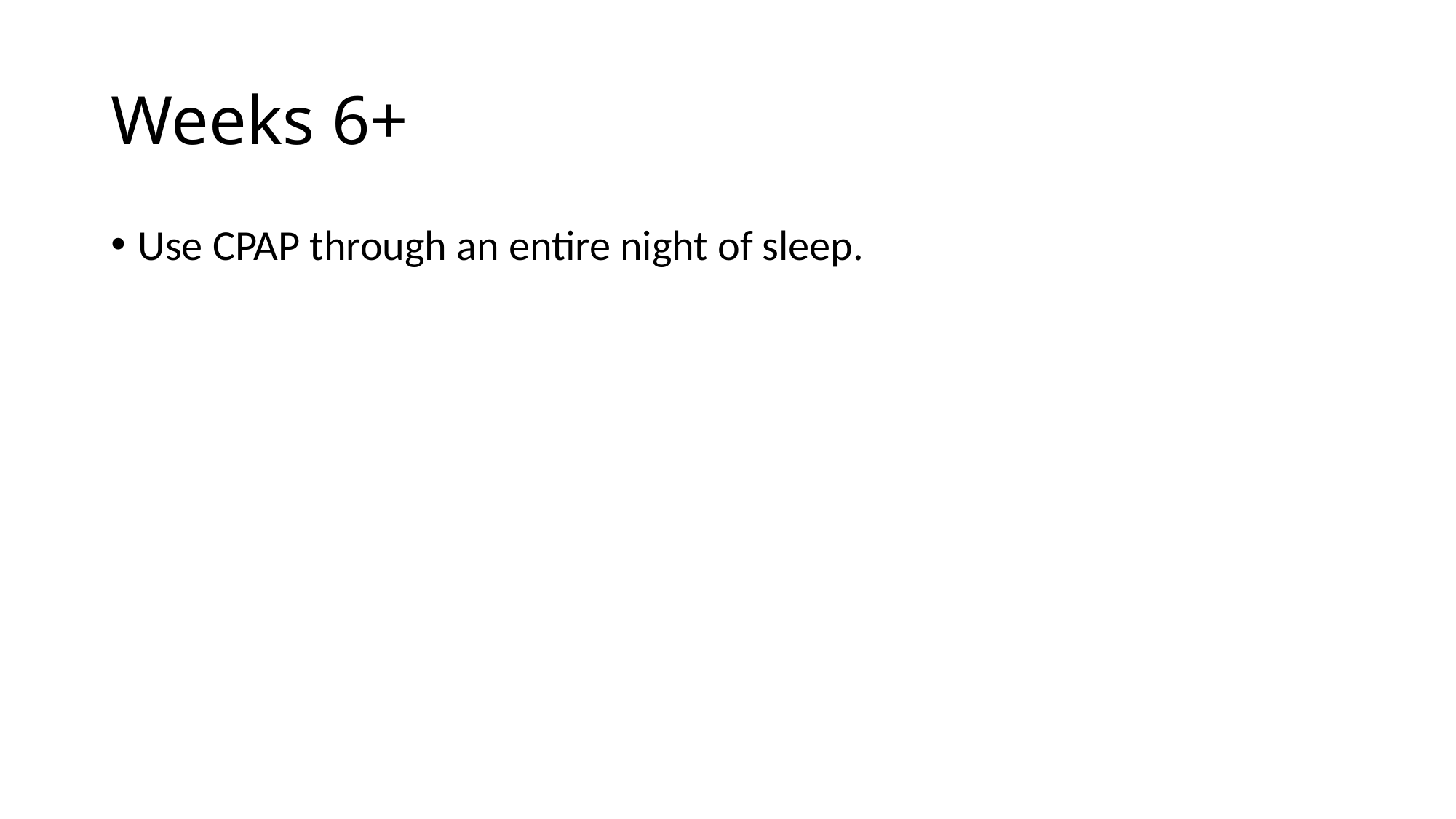

# Weeks 6+
Use CPAP through an entire night of sleep.

## Slide 20
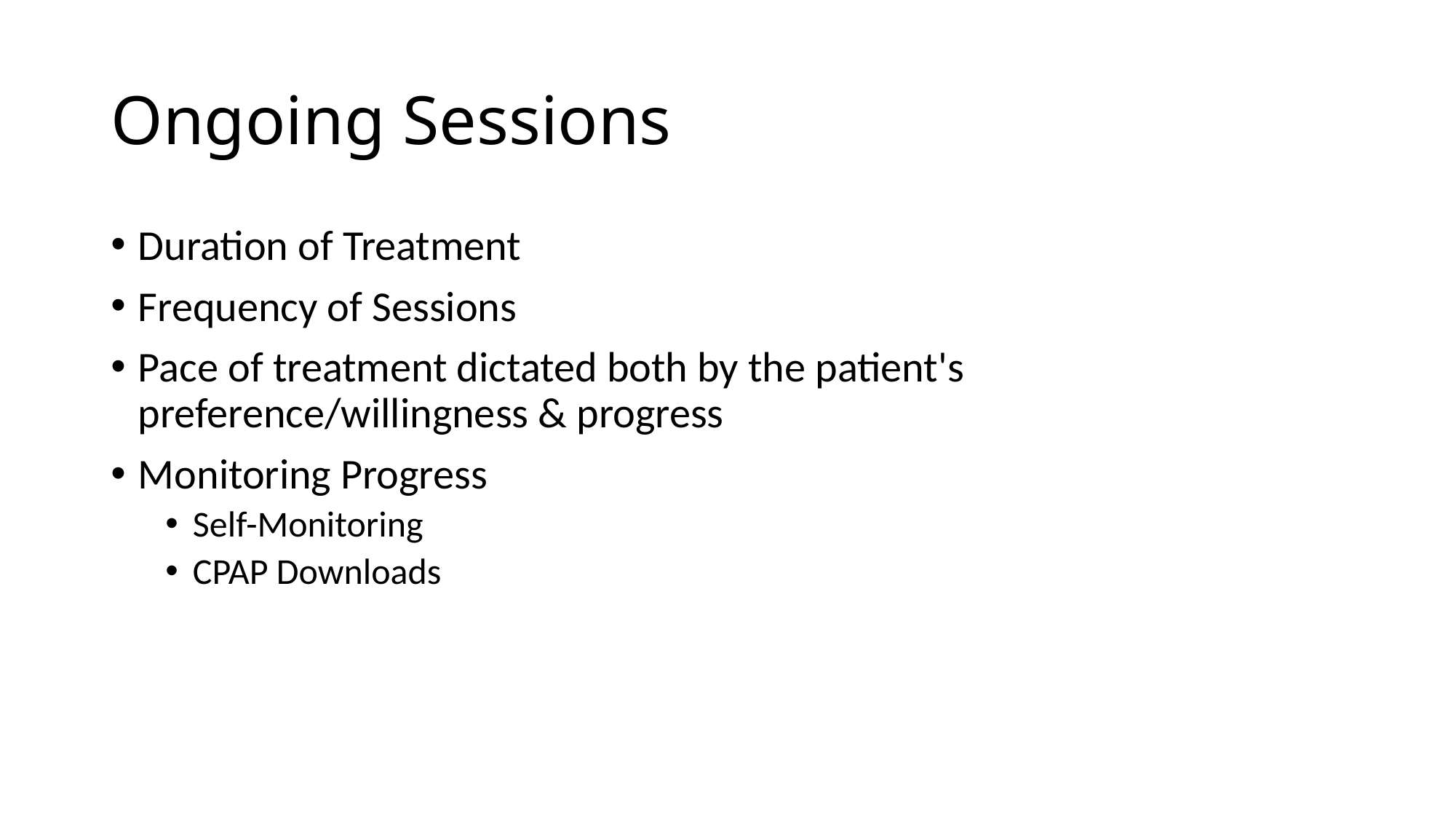

# Ongoing Sessions
Duration of Treatment
Frequency of Sessions
Pace of treatment dictated both by the patient's preference/willingness & progress
Monitoring Progress
Self-Monitoring
CPAP Downloads

## Slide 21
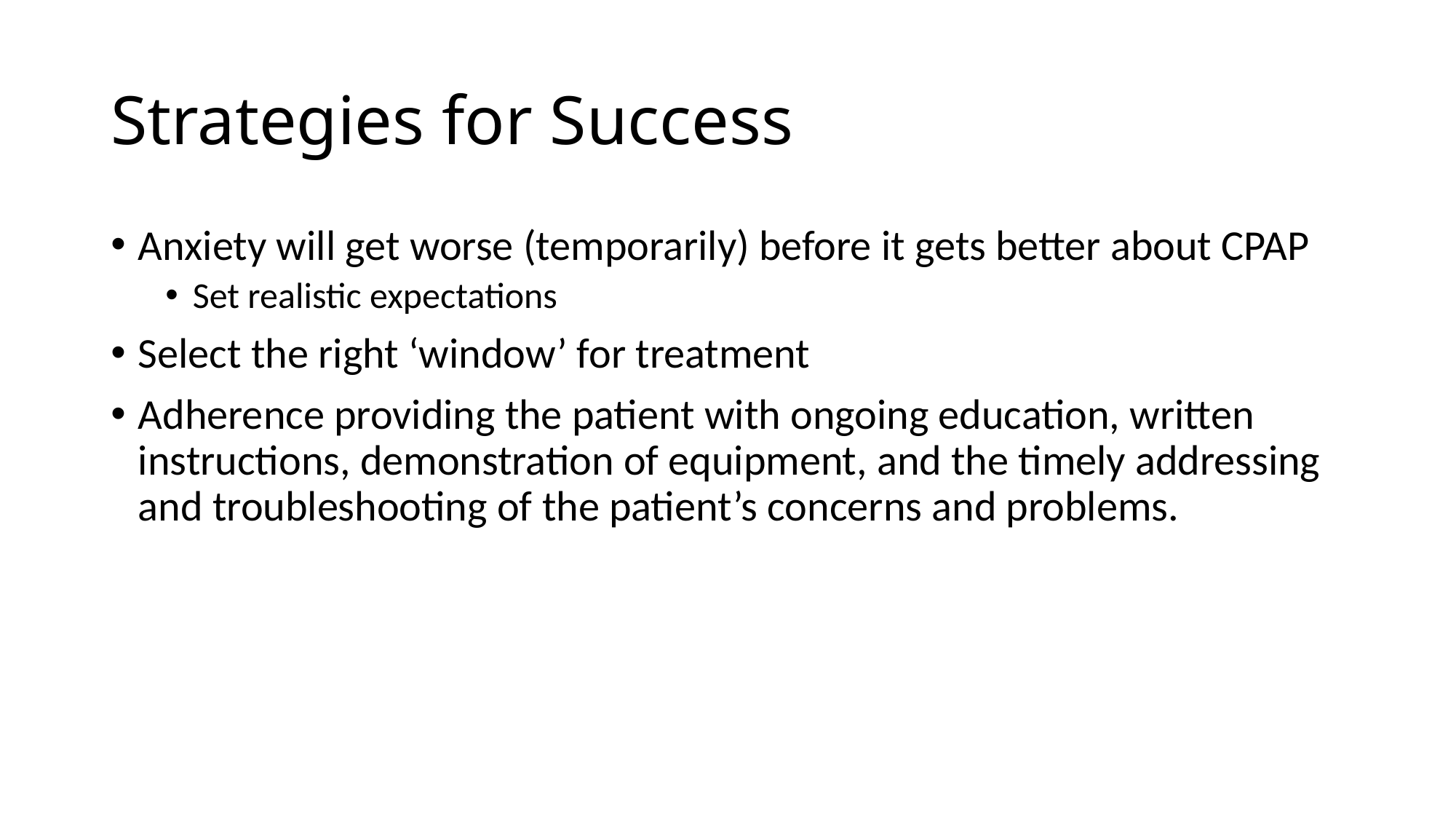

# Strategies for Success
Anxiety will get worse (temporarily) before it gets better about CPAP
Set realistic expectations
Select the right ‘window’ for treatment
Adherence providing the patient with ongoing education, written instructions, demonstration of equipment, and the timely addressing and troubleshooting of the patient’s concerns and problems.

## Slide 22
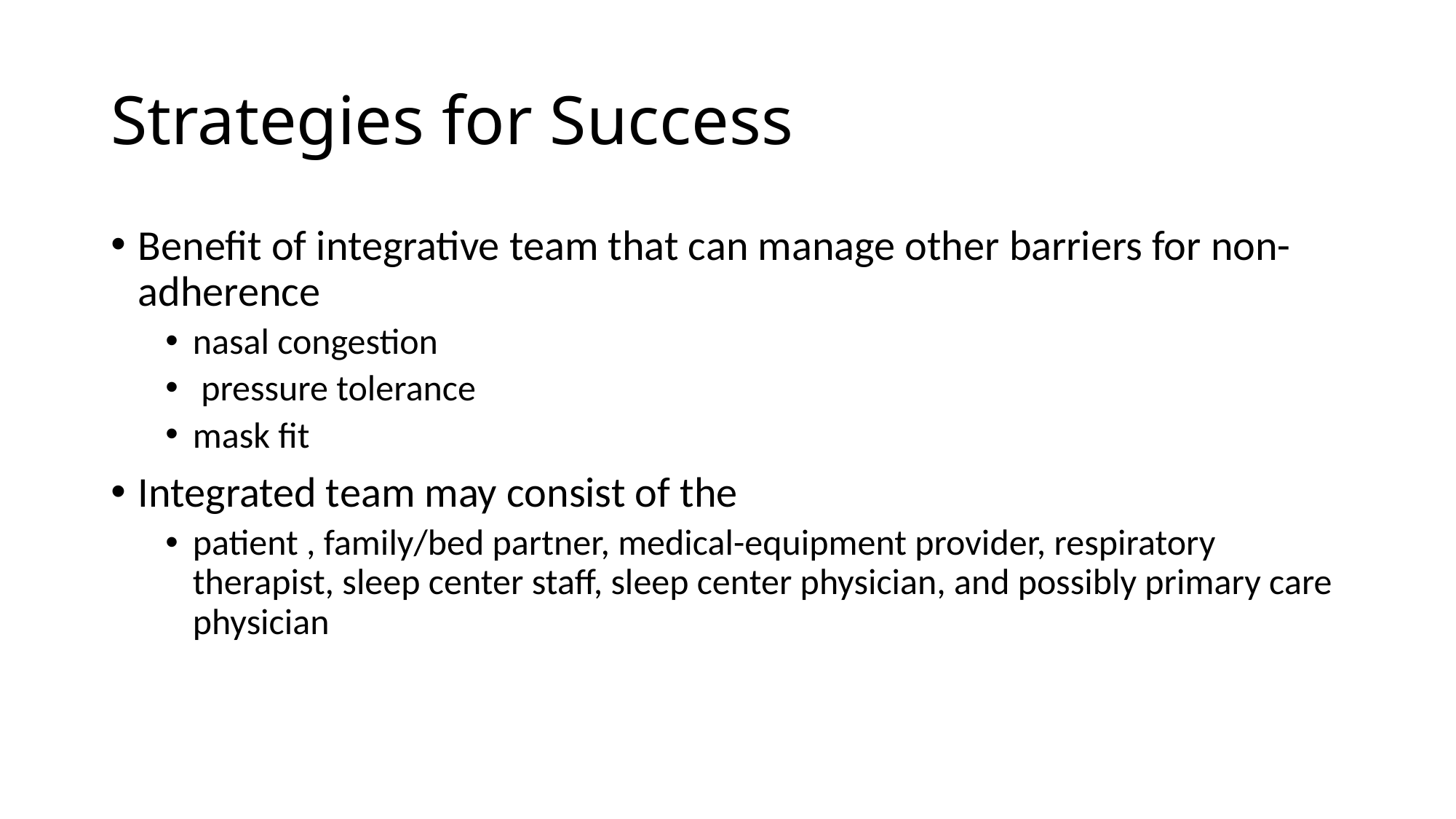

# Strategies for Success
Benefit of integrative team that can manage other barriers for non-adherence
nasal congestion
 pressure tolerance
mask fit
Integrated team may consist of the
patient , family/bed partner, medical-equipment provider, respiratory therapist, sleep center staff, sleep center physician, and possibly primary care physician

## Slide 23
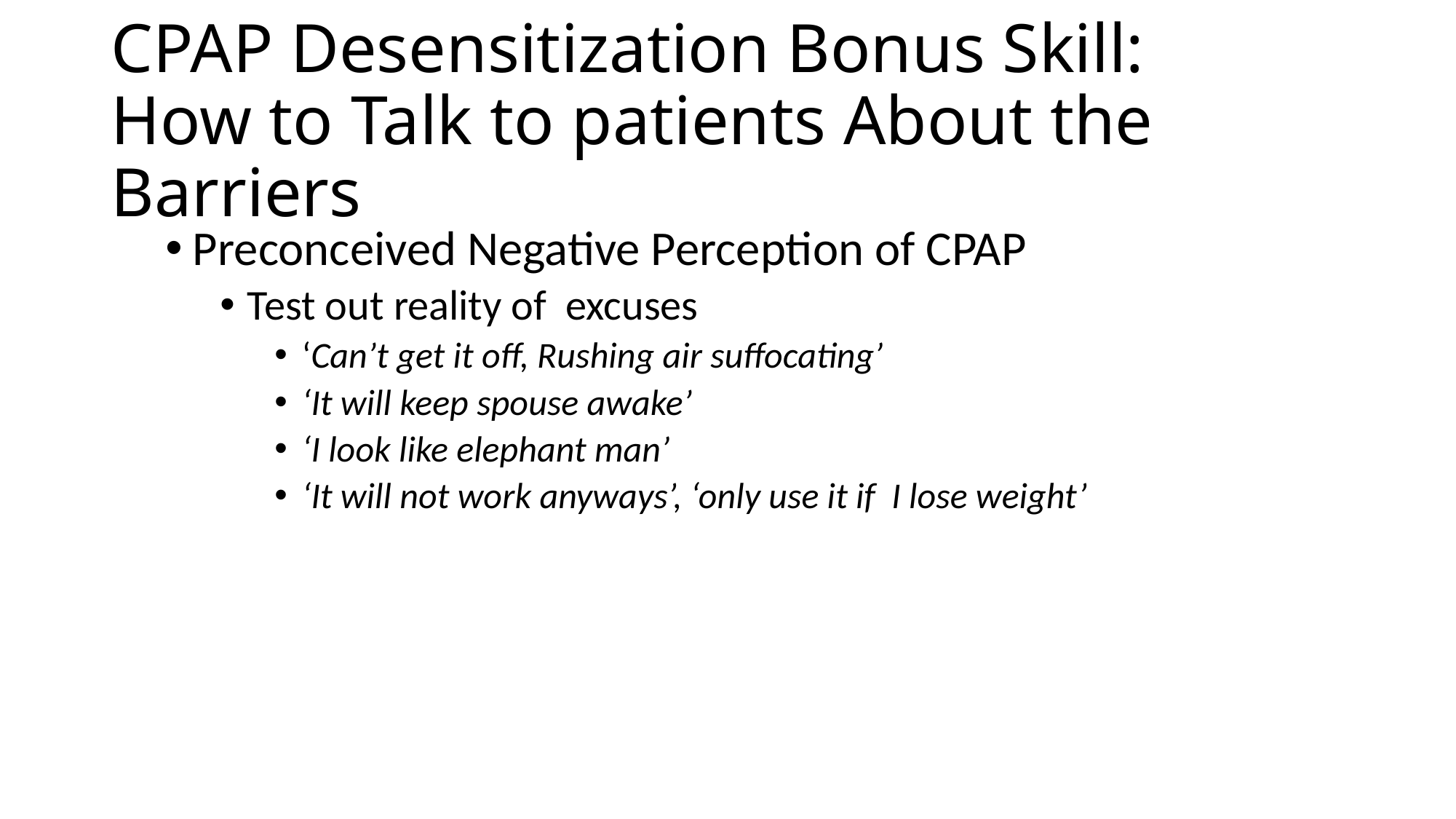

# CPAP Desensitization Bonus Skill: How to Talk to patients About the Barriers
Preconceived Negative Perception of CPAP
Test out reality of excuses
‘Can’t get it off, Rushing air suffocating’
‘It will keep spouse awake’
‘I look like elephant man’
‘It will not work anyways’, ‘only use it if I lose weight’

## Slide 24
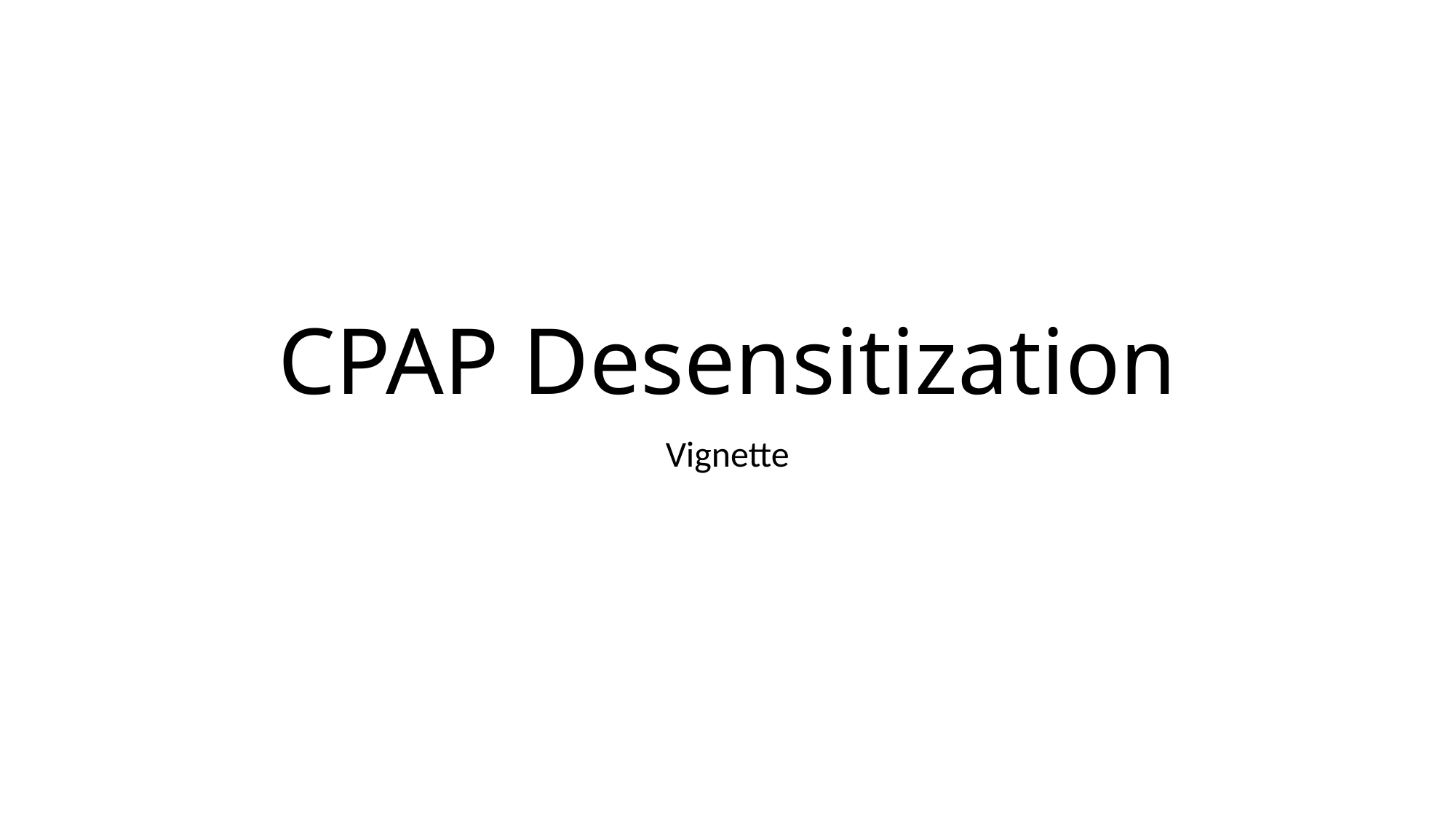

# CPAP Desensitization
Vignette

## Slide 25
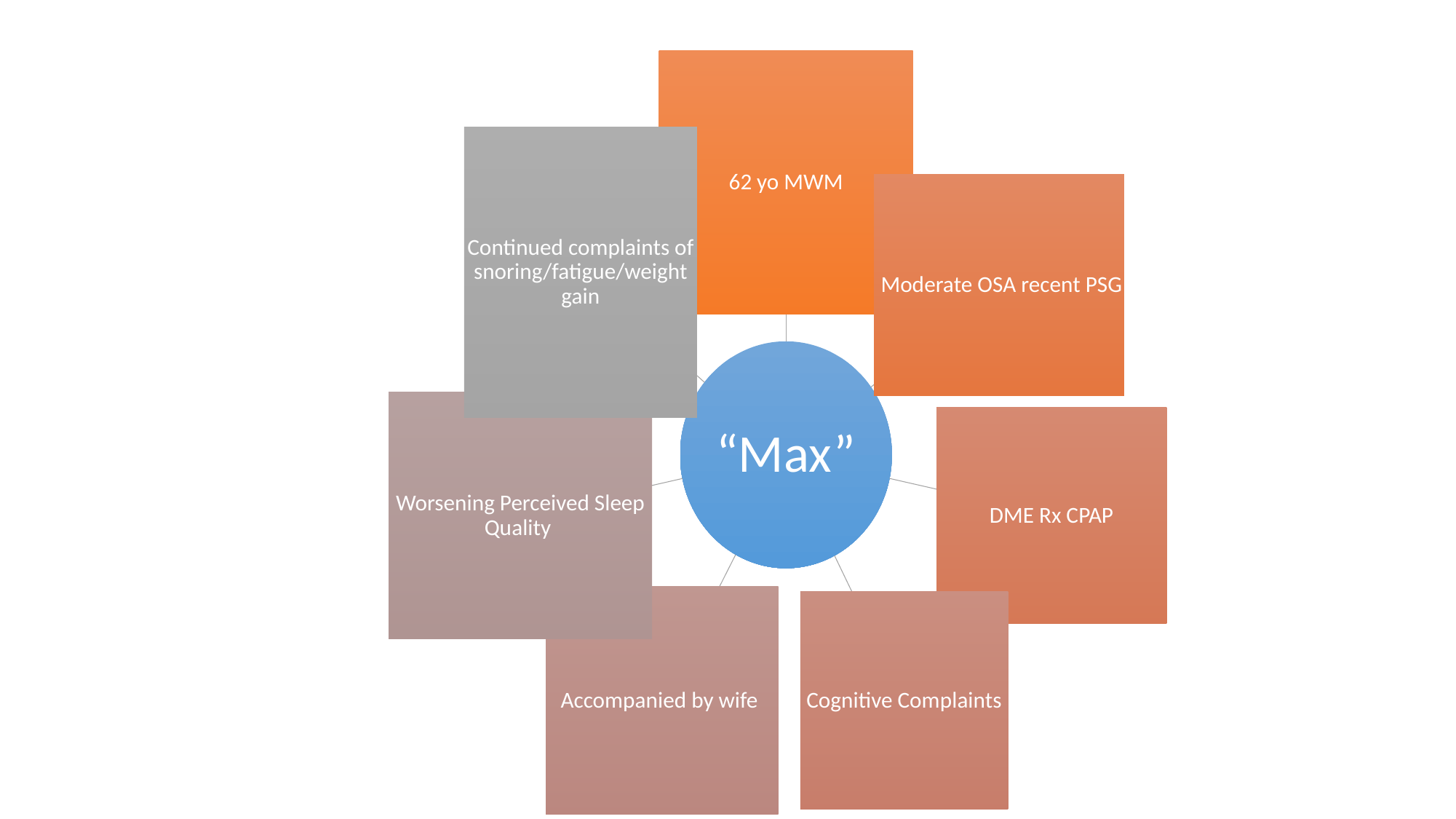

## Slide 26
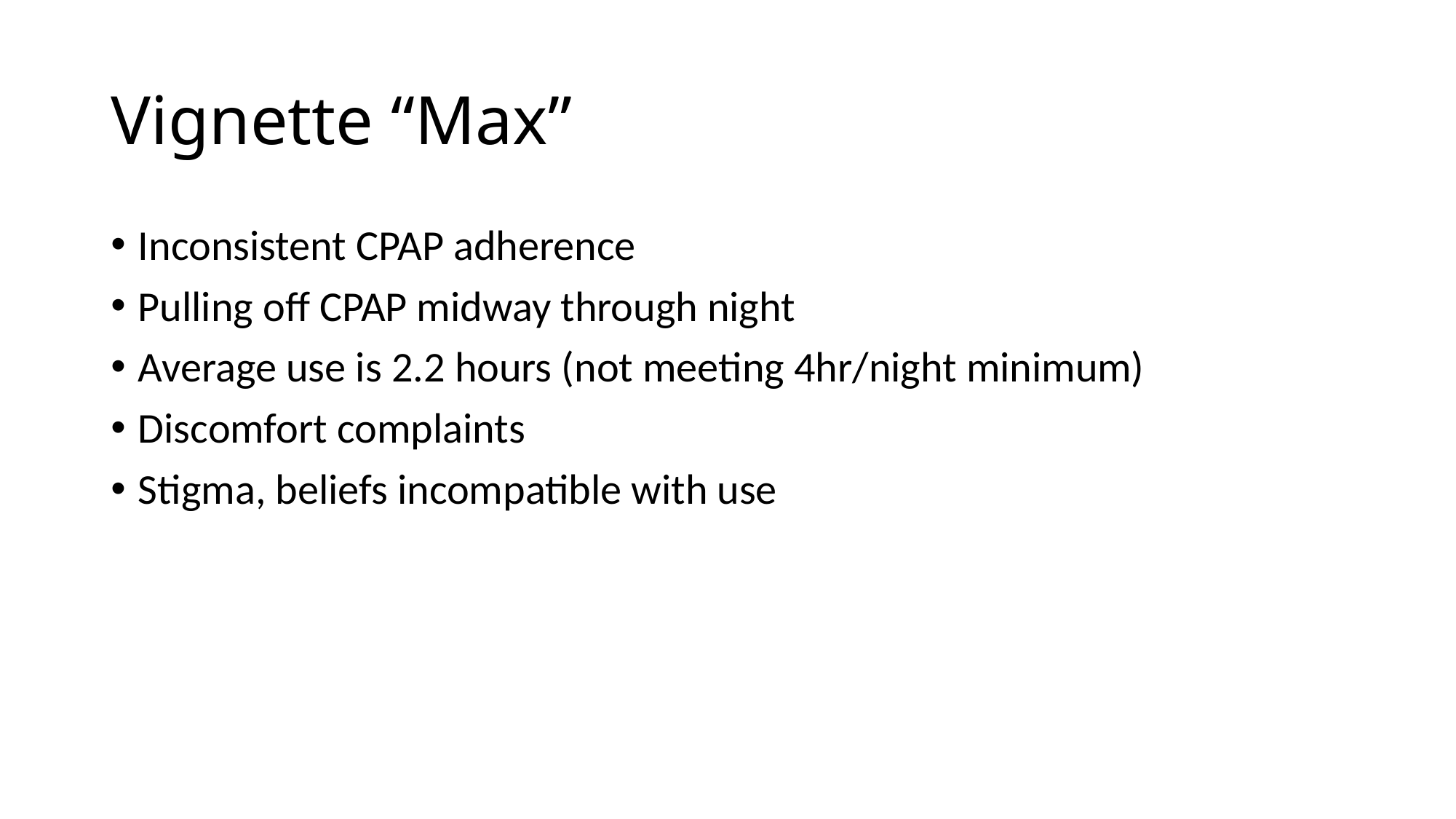

# Vignette “Max”
Inconsistent CPAP adherence
Pulling off CPAP midway through night
Average use is 2.2 hours (not meeting 4hr/night minimum)
Discomfort complaints
Stigma, beliefs incompatible with use

## Slide 27
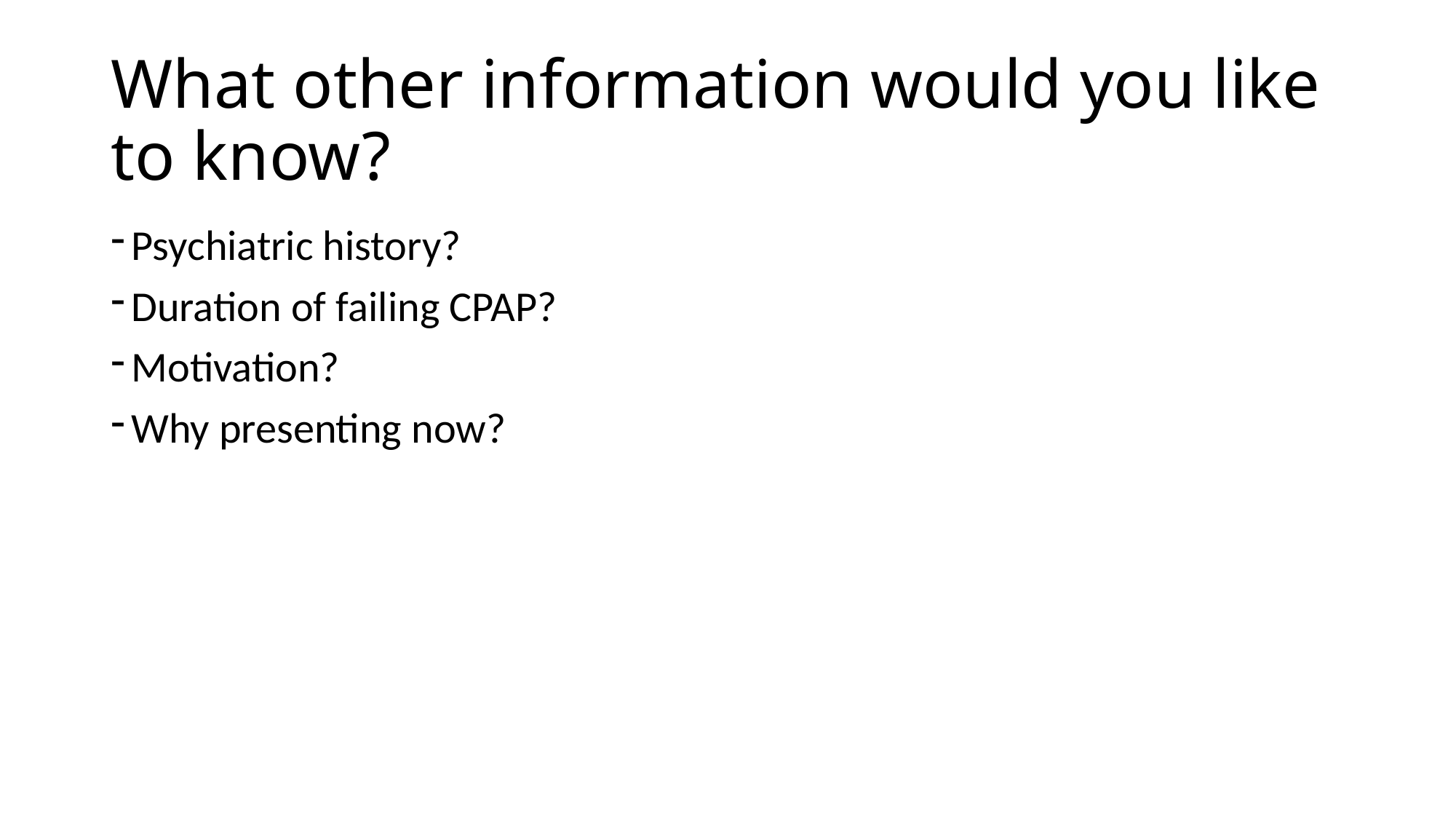

# What other information would you like to know?
Psychiatric history?
Duration of failing CPAP?
Motivation?
Why presenting now?

## Slide 28
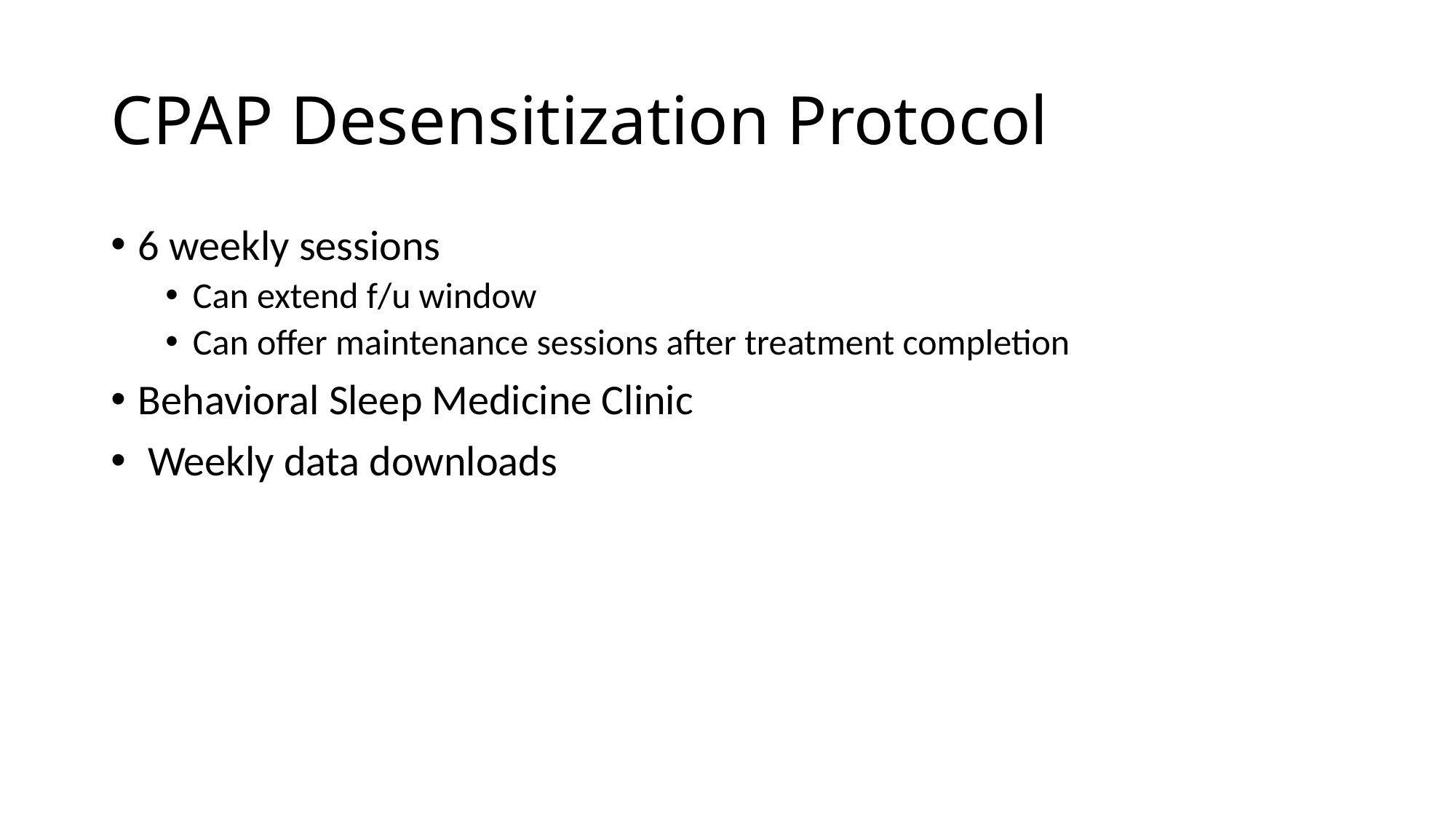

# CPAP Desensitization Protocol
6 weekly sessions
Can extend f/u window
Can offer maintenance sessions after treatment completion
Behavioral Sleep Medicine Clinic
 Weekly data downloads

## Slide 29
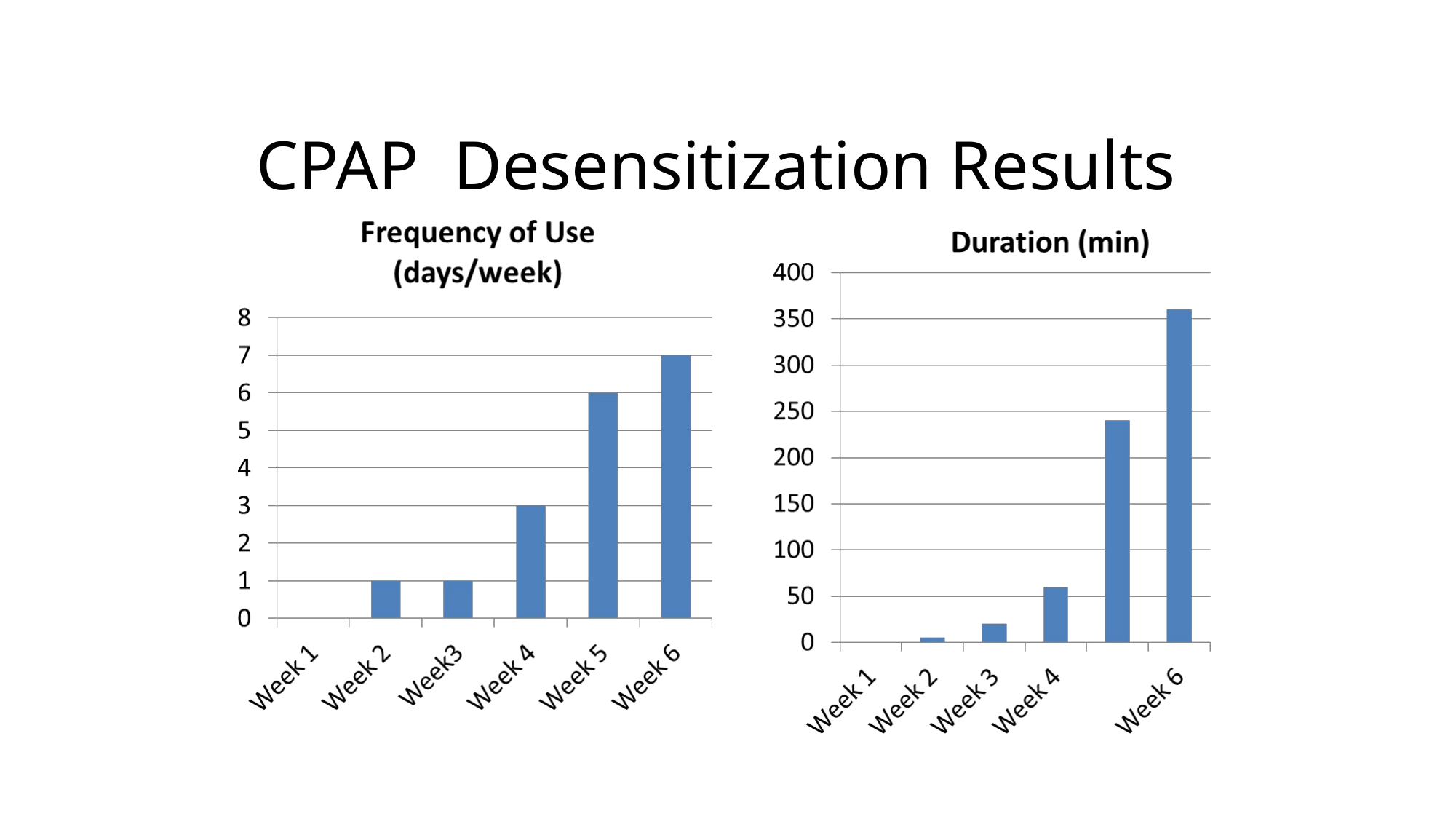

# CPAP Desensitization Results

## Slide 30
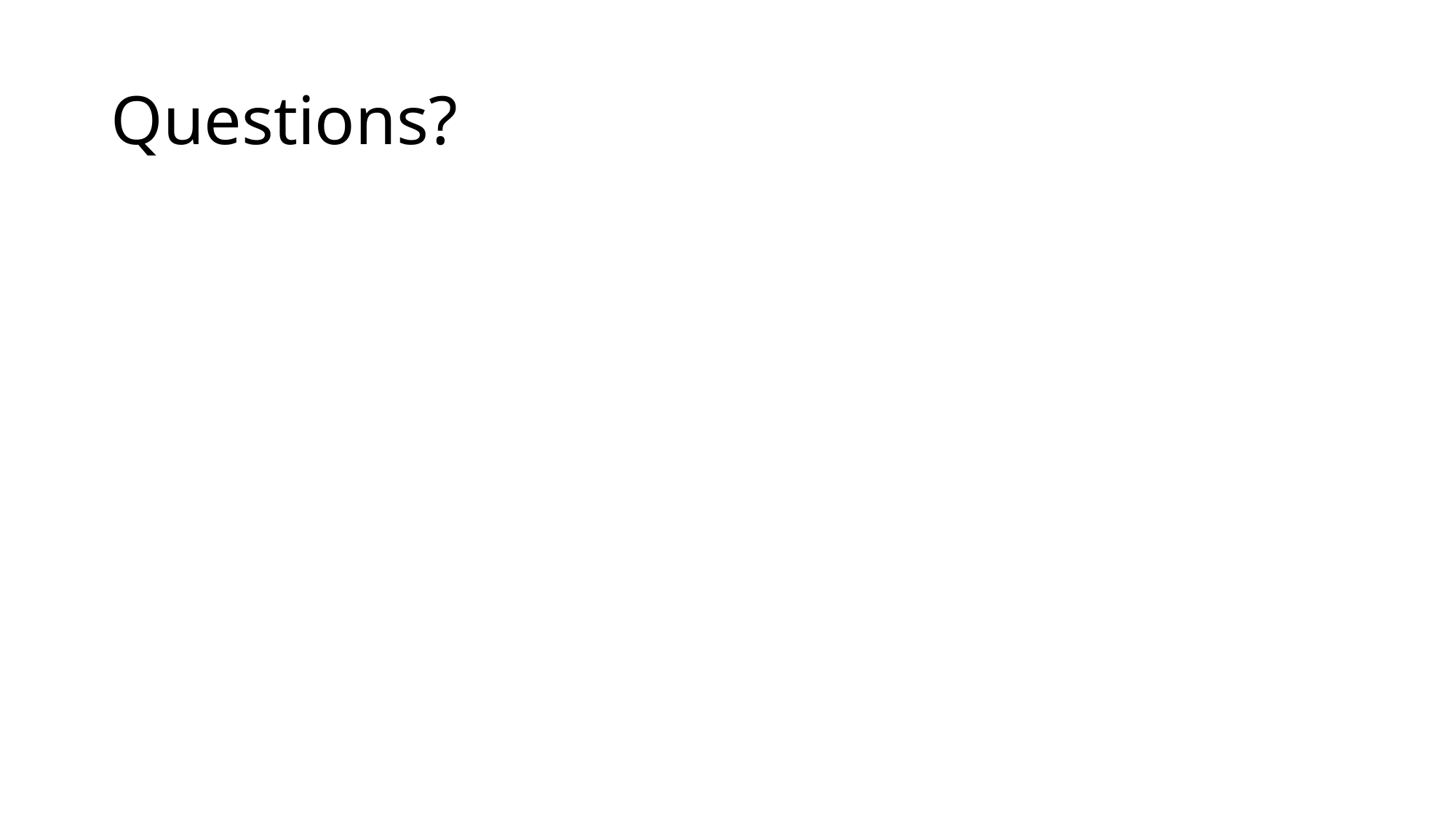

# Questions?
