# Supplementary material for: Improving CPAP Adherence for Obstructive Sleep Apnea: A Practical Application Primer on CPAP Desensitization
Source: MedEdPORTAL. 2020 Sep 15;16:10963. doi: 10.15766/mep_2374-8265.10963 (PMC7499811; doi:10.15766/mep_2374-8265.10963)
Supplement: Supplementary file 1 — CPAP Desensitization.pptxCPAP Interactive Role-Play.docxCPAP Desensitization Patient Protocol.docxCPAP Pre- & Posttest.docx [file mep_2374-8265.10963-s001.zip › B. CPAP Interactive Role-play.docx]

**CPAP Desensitization Interactive Role-Play**

Preparation:

- Obtain either a sanitized CPAP mask and/or machine from sleep lab for workshop purposes

OR

- Use paper lunch bag with narrow slits at the bottom to mimic shallow breathing/claustrophobia reaction

Step 1. (5-10 minutes) Allow learners to experience sensations of wearing/ breathing via a CPAP mask/paper bag to imitate the feeling of claustrophobia or suffocation some patients may experience, noticing the sensations, which may include:

- Discomfort/Pain
- Breathing rate/heart rate/BP/sweat conductance
- Monitor thoughts/feelings/reactions
- If CPAP mask/equipment is available for the workshop, additional experiential exposure should be tried including:
  - Assembly of mask/hose/CPAP equipment
  - Placing of CPAP mask on oneself with proper adjustment and check for proper mask fit/seal
  - Operation of CPAP equipment
  - Sensation of CPAP equipment when the pressure is turned on and up to a pressure setting 10+
- *Depending on number of learners, turns can be taken using CPAP mask/equipment as time allows. With 3+ learners, the paper lunch bag obtained in larger quantities can be utilized to optimize workshop time management*

Discussion questions:

1. What emotions would be associated with the above noticed sensations?
2. How would this impact ability to relax at night time or sleep? Go back to sleep in middle of the night?
3. What would it be like if bed-partner there to observe it?

Step 2. (15 minutes) The workshop leader ‘clinician’ role-plays a desensitization exercise with a learner as ‘patient’

- The ‘patient’ is asked to act as if their discomfort with the CPAP is of a moderate-severe intensity
- The ‘clinician’ observes the patient wearing the mask, noticing any signs of discomfort or anxiety
- Practice relaxation exercise while the patient is wearing the CPAP/breathing into the bag using script or audio recording (widely available on youtube.com or Calm App)
- In session exposure-therapy exercise is conducted for a duration of 5-10 minutes during which time the ‘clinician’ coaches the ‘patient’ to notice their anxiety decreasing to a tolerable level over the duration of the exercise

Step 3. (Optional, 15 minutes) Repeat Step 2 with groups of 2 learners, 1 acting as ‘clinician’ and 1 as the ‘patient’. Preceptor provides observation, guidance, and feedback rotating around the pairs of learners
