# Supplementary material for: Improving CPAP Adherence for Obstructive Sleep Apnea: A Practical Application Primer on CPAP Desensitization
Source: MedEdPORTAL. 2020 Sep 15;16:10963. doi: 10.15766/mep_2374-8265.10963 (PMC7499811; doi:10.15766/mep_2374-8265.10963)
Supplement: Supplementary file 1 — CPAP Desensitization.pptxCPAP Interactive Role-Play.docxCPAP Desensitization Patient Protocol.docxCPAP Pre- & Posttest.docx [file mep_2374-8265.10963-s001.zip › C. CPAP Desensitization Patient Protocol.docx]

**CPAP Desensitization**

*Patient Protocol*

**Prior to Session 1:**

1. Try multiple masks if necessary and choose the CPAP mask that makes you feel most comfortable
2. Avoid switching around to different masks once you have selected one.
3. Learn how your CPAP machine works, maintain it properly, and ensure ramp/humidifier working.

**Sessions 1-2**

1. Hold mask over your nose, and practice breathing while awake. While you are doing this, keep your mouth closed and breathe regularly through your nose. Start with short periods of time (5-15 min) and gradually build up to longer periods of time.
2. Do relaxing activities or try to take a nap with the CPAP mask on.
3. Do not try wearing CPAP during sleep until you are comfortable with it during the daytime.
4. Create Fear Hierarchy (Individualized, see sample below)
5. Introduce and practice Relaxation Training (15 min daily with or without CPAP)

**Sessions 3-4**

1. Turn the CPAP airflow ON and wear the mask over your nose with the straps on your head. Practice breathing with CPAP on while awake. If your machine has a RAMP button, patient may use this function to keep the pressure at a low level during practices. Wear CPAP for longer periods of time until you can have it on for 15–20 min comfortably.
2. Wear the mask at home, without the CPAP machine, while awake for up to one hour each day

**Sessions 5-6**

1. Wear CPAP at night when you go to sleep.
2. Use CPAP during initial 3-4 hours of nocturnal sleep at first, even if you end up pulling it off or needing to take it off.

**Sessions 6+**

1. Use CPAP through an entire night of sleep.

Example of a Fear Hierarchy Diagram to follow a desensitization protocol on:


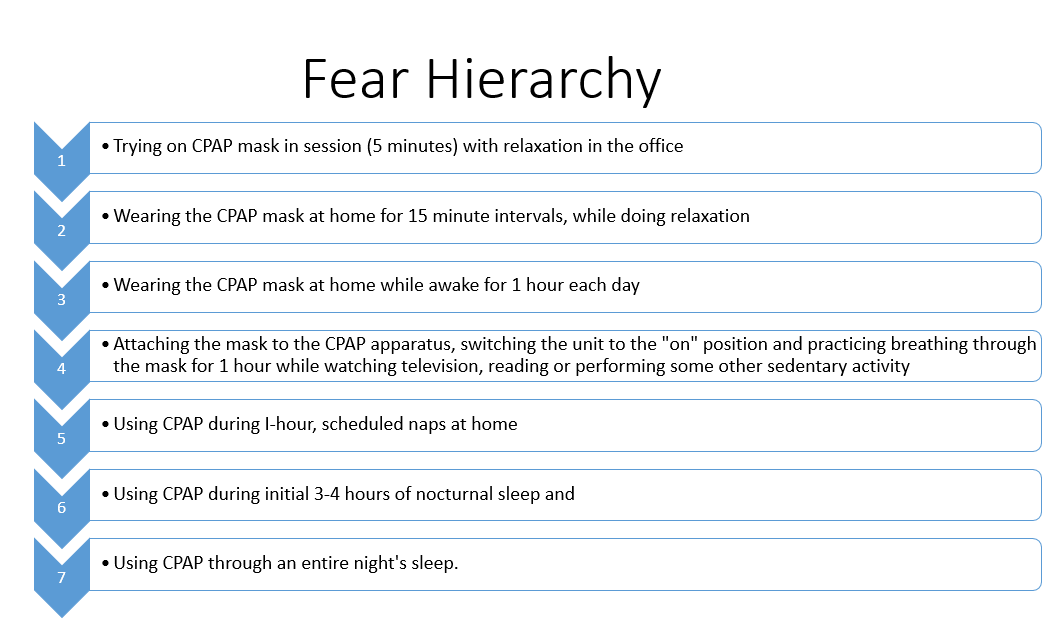


**Fear Hierarchy can be personalized to each patient depending on where they are starting out in their comfort level with their CPAP, how quickly they can tolerate progression through the steps, and how compliant they are*
