# Supplementary material for: Improving CPAP Adherence for Obstructive Sleep Apnea: A Practical Application Primer on CPAP Desensitization
Source: MedEdPORTAL. 2020 Sep 15;16:10963. doi: 10.15766/mep_2374-8265.10963 (PMC7499811; doi:10.15766/mep_2374-8265.10963)
Supplement: Supplementary file 1 — CPAP Desensitization.pptxCPAP Interactive Role-Play.docxCPAP Desensitization Patient Protocol.docxCPAP Pre- & Posttest.docx [file mep_2374-8265.10963-s001.zip › D. CPAP Pre- & Posttest.docx]

**Student Evaluation**

**TOPIC: Date: Learner Type:**

**PRE-TEST**

Please rate your level of agreement with the following statements:

|  |  | **1**  Strongly Disagree | **2**  Disagree | **3**  Neutral | **4**  Agree | **5**  Strongly Agree |
| --- | --- | --- | --- | --- | --- | --- |
| 1 | This is an important topic for a clinical workshop |  |  |  |  |  |
| 2 | I understand clinical development of this topic |  |  |  |  |  |
| 3 | I understand the illness surrounding this topic |  |  |  |  |  |
| 4 | I feel comfortable evaluating/diagnosing on this topic |  |  |  |  |  |
| 5 | I have the skills to evaluate/diagnose on this topic |  |  |  |  |  |
| 6 | I feel comfortable solving clinical problems on this topic |  |  |  |  |  |
| 7 | I have the skills to solve clinical problems on this topic |  |  |  |  |  |
| 8 | I am enthusiastic about applying/practicing this clinical skill |  |  |  |  |  |
| 9 | I will encounter many opportunities to use this clinical skill |  |  |  |  |  |
| 10 | I would like to seek out more opportunities to use this clinical skill |  |  |  |  |  |

**Student Evaluation**

**TOPIC: Date: Learner Type:**

**POST-TEST**

Please rate your level of agreement with the following statements:

|  |  | **1**  Strongly Disagree | **2**  Disagree | **3**  Neutral | **4**  Agree | **5**  Strongly Agree |
| --- | --- | --- | --- | --- | --- | --- |
| 1 | This is an important topic for a clinical workshop |  |  |  |  |  |
| 2 | I understand clinical development of this topic |  |  |  |  |  |
| 3 | I understand the illness surrounding this topic |  |  |  |  |  |
| 4 | I feel comfortable evaluating/diagnosing on this topic |  |  |  |  |  |
| 5 | I have the skills to evaluate/diagnose on this topic |  |  |  |  |  |
| 6 | I feel comfortable solving clinical problems on this topic |  |  |  |  |  |
| 7 | I have the skills to solve clinical problems on this topic |  |  |  |  |  |
| 8 | I am enthusiastic about applying/practicing this clinical skill |  |  |  |  |  |
| 9 | I will encounter many opportunities to use this clinical skill |  |  |  |  |  |
| 10 | I would like to seek out more opportunities to use this clinical skill |  |  |  |  |  |
| 11 | This workshop was well organized |  |  |  |  |  |
| 12 | I found this small group format useful |  |  |  |  |  |
| 13 | The facilitators effectively conducted this exercise |  |  |  |  |  |
| 14 | This format was valuable for learning this topic |  |  |  |  |  |
| 15 | The facilitator appeared knowledgeable on this topic |  |  |  |  |  |
| 16 | The interactive component of this topic was useful |  |  |  |  |  |
| 17 | Audio/Visual Aids Reinforced the content |  |  |  |  |  |

1. What did you think were the strengths of this workshop?
2. What did you think were some weaknesses regarding this workshop? What would you change?

**Overall, how would you rate this lecture (please circle):**

| 1 Excellent | 2 Very Good | 3 Good | 4 Fair | 5 Poor |
| --- | --- | --- | --- | --- |
